# The immune checkpoints storm in COVID‐19: Role as severity markers at emergency department admission

**DOI:** 10.1002/ctm2.573

**Published:** 2021-10-18

**Authors:** José Avendaño‐Ortiz, Roberto Lozano‐Rodríguez, Alejandro Martín‐Quirós, Verónica Terrón, Charbel Maroun‐Eid, Karla Montalbán‐Hernández, Jaime Valentín‐Quiroga, Miguel Ángel García‐Garrido, Elena Muñoz del Val, Álvaro del Balzo‐Castillo, María Peinado, Laura Gómez, Carmen Herrero‐Benito, Carolina Rubio, José Carlos Casalvilla‐Dueñas, Paloma Gómez‐Campelo, Alejandro Pascual‐Iglesias, Carlos del Fresno, Luis A. Aguirre, Eduardo López‐Collazo

**Affiliations:** ^1^ The Innate Immune Response Group, IdiPAZ La Paz University Hospital Madrid Spain; ^2^ Tumor Immunology Laboratory IdiPAZ, La Paz University Hospital Madrid Spain; ^3^ Emergency Department and Emergent Pathology Research Group IdiPAZ La Paz University Hospital Madrid Spain; ^4^ Biobank Platform, IdiPAZ La Paz University Hospital Madrid Spain; ^5^ CIBER of Respiratory Diseases (CIBERES) Madrid Spain

Dear Editor,

Despite the improvement in prophylaxis by vaccination, severe acute respiratory syndrome coronavirus 2 (SARS‐CoV‐2) infection and its associated pathology, COVID‐19, still constitute a challenge. Clinicians lack effective treatments and robust predictors to identify patients who will develop severe disease during their stay. The role of cytokines (e.g., IL‐6) as biomarkers and therapeutic targets was evaluated at the beginning of the pandemic. However, their relevance are still being discussed.^1^ Patients with severe COVID‐19 show an impairment of the immune system allowing secondary infections,^2^, ^3^, ^4^, ^5^ and numerous studies have found how T cell polyfunctionality decreases.^5^, ^6^ In this regard, immune checkpoints (ICs), a family of molecules known for their ability to modulate immune response and induce T cell exhaustion and apoptosis, become interesting not only as potential early biomarkers of patients’ evolution but also as possible pharmaceutical targets.^7^

Thus, we recruited 69 patients and 15 healthy volunteers (HVs) from the emergency department (ED) of La Paz University Hospital. We analysed nine ICs on admission, previous to any treatment, in plasma from patients classified according to their outcome: mild (outpatients + hospitalized with no O_2_ requirement, *n* = 29), severe (hospitalized with O_2_ requirement, *n* = 26) and deceased (exitus, *n* = 14; 28‐day mortality according WHO U07.1 code) (Figure 1A,B and Supplemental Figure S1 and Table S1). We found sCD25, sTim‐3, Galectin‐9, sPD‐L1 and sCD86 exhibited differences between groups (Figure 1C–L). sCD25, sTim‐3 and Galectin‐9 showed to be increased in patients versus HV despite the fact that they developed mild disease (Figure 1C–E). In addition, sCD25, sTim‐3, Galectin‐9, sPD‐L1 and sPD‐1 showed increased levels in severe patients and/or exitus compared with mild ones (Figure 1C–G). In contrast, the co‐stimulatory molecule CD86 (sCD86) exhibited lower levels in mild and exitus patients compared with severe ones (Figure 1H). Levels of other ICs (sLAG‐3, sCTLA‐4 and sCD137) are shown in Figure 1I–L. Note that similar results were obtained when patients were classified according to a multi‐organ failure score (qSOFA) (Supplemental Figure S2). In line with this, IC expression on T lymphocytes was also evaluated (Supplemental Figures S3 and S4).

**FIGURE 1:**
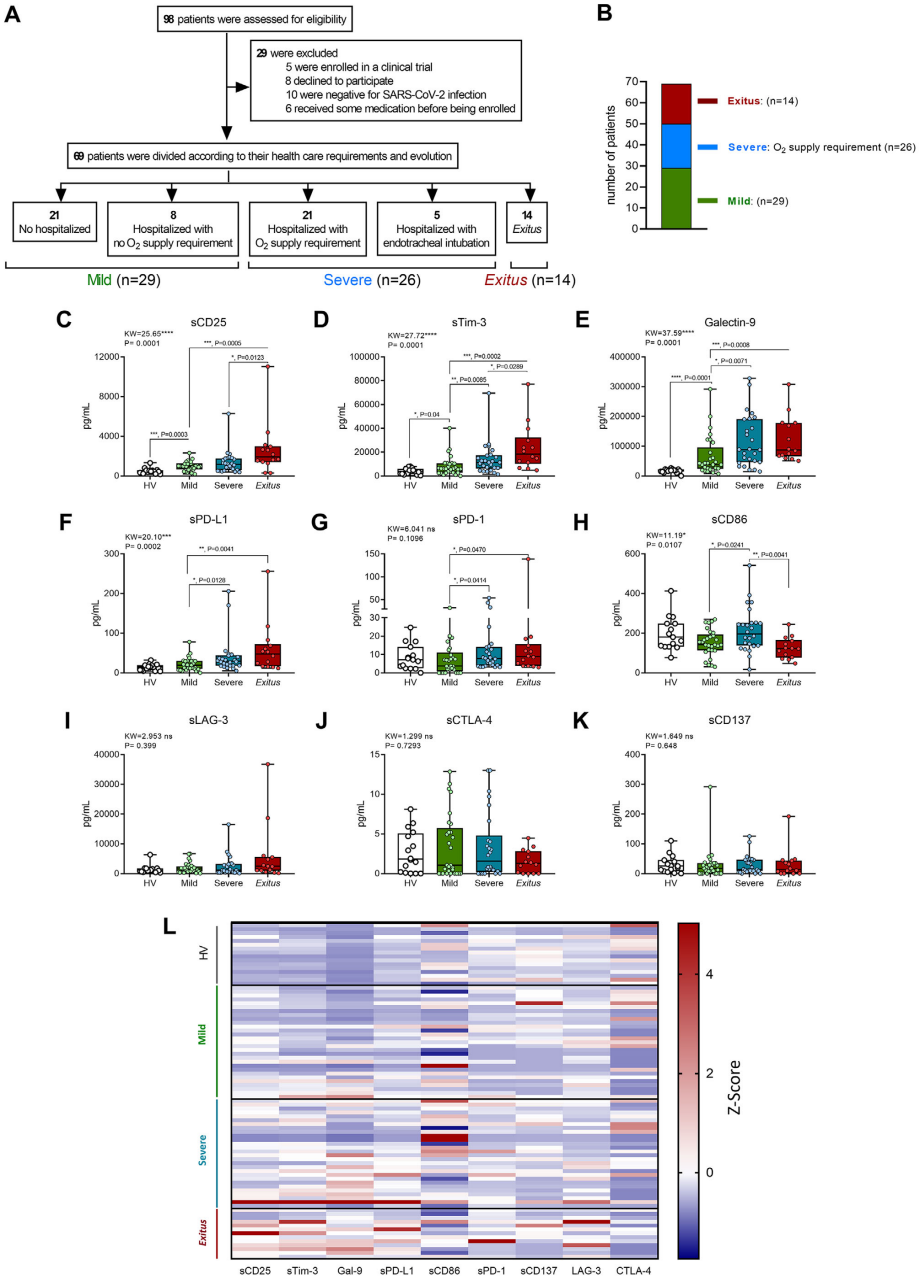
Distribution of patients and plasma immune checkpoint levels according to outcome of patients with COVID‐19 on admission, compared with HVs. (A) and (B) Distribution of patients with COVID‐19 in the severity groups according to evolution and their oxygen requirement during their stay: mild (*n* = 29); severe (*n* = 26); and exitus (*n* = 14). Quantification of plasma soluble immune checkpoints according to disease severity of patients with COVID‐19 on admission compared with HVs: sCD25 (C), sTim‐3 (D), Galectin‐9 (E), sPD‐L1 (F), sPD‐1 (G), sCD86 (H), sLAG‐3 (I), sCTLA‐4 (J) and sCD137 (K). (L) Heatmap of plasma immune checkpoints levels *Z*‐score of HVs and patients with COVID‐19 on admission according to the severity group: mild (1); severe (2); and exitus (3) is shown. (C–D) Data are shown as pg/mL and were analysed by Kruskal–Wallis and Mann–Whitney U tests. Data are presented in box‐and‐whisker plots (min to max). *p < .05; **p < .01; ***p < .001; ****p < .0001; KW, Kruskal–Wallis statistic

Next, we focused on the five ICs with significant differences between groups. sCD25, sTim‐3, Galectin‐9, and sPD‐L1 showed a positive correlation with severity (Supplemental Figure S5). sCD86 did not show a patent trend (Supplemental Figure S5G). Severity in COVID‐19 patients has been associated with differences in sex, age and comorbidities.^8^, ^9^, ^10^ Nonetheless, we did not find significant differences in IC levels between genders (Supplemental Figure S6) and only slight differences within comorbidities (Supplemental Figure S7). However, levels of both sCD25 and sTim‐3 on admission not only correlated with a poor prognosis but also with age (Supplemental Figure S8). Other correlations are shown in Supplemental Figure S9.

We moved to study the changes in ICs throughout the hospital stay until discharge or exitus. Despite initial similarities between severe and exitus groups, their evolutions were disparate (Figure 2). Levels of sCD25, sTim‐3 and Galectin‐9 for mild and severe patients showed a trend toward the levels reported for HVs, while exitus exhibited a progressive elevation (Figure 2, right panels). Levels of sCD86 increased or levelled off in patients in the mild and severe groups, in contrast to the decrease observed in exitus (Figure 2E). Similar results were obtained when the analysis was performed using days from onset of symptoms (Supplemental Figure S10).

**FIGURE 2:**
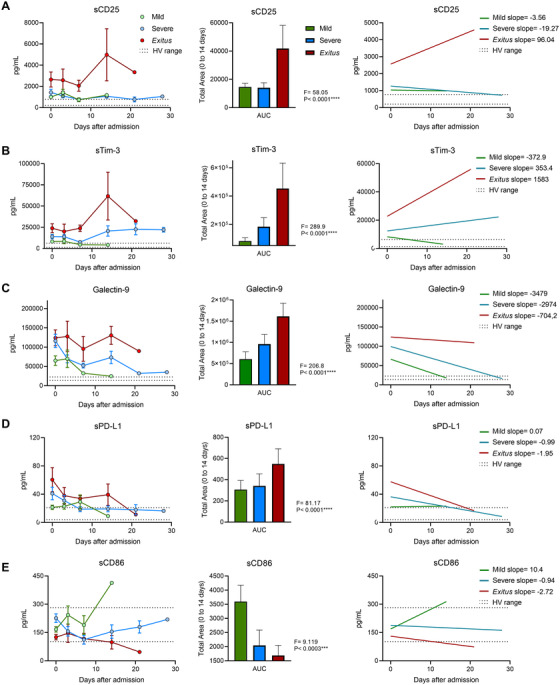
Longitudinal analysis of plasma immune checkpoint levels of patients with COVID‐19 according to their evolution and hospital needs from admission. Longitudinal concentrations of plasma sCD25 (A), sTim‐3 (B), Galectin‐9 (C), sPD‐L1 (D) and sCD86 (E) in patients with COVID‐19 according to the severity group from admission are shown (left panels). Total area under the curves (AUCs) from 0 to 14 days after admission of longitudinal concentrations of plasma sCD25 (A), sTim‐3 (B), Galectin‐9 (C), sPD‐L1 (D), and sCD86 (E) of patients with COVID‐19 according to the severity group on admission are shown (central panels). Linear regression and curve slopes are shown in right panels. Dashed lines show the HV ranges of plasma immune checkpoint levels. Data are pg/mL concentrations, and subgroup differences were analysed by the one‐way analysis of variance. Data represent mean ± SEM. ***p < 0.001; ****p < 0.0001; *F*, *F*‐statistic

To explore the possible role of ICs in both the T cell exhaustion and lymphocytopenia reported in patients with COVID‐19,^4^, ^5^ we calculated the correlation between absolute lymphocyte count (ALC) and levels of circulating ICs on admission. Levels of sCD25, sTim‐3, Galectin‐9 and sPD‐L1, but not sCD86, showed a negative correlation with ALC (Supplemental Figures S11A and S12). Moreover, negative correlation between T cell proliferation and circulating levels of sCD25, sTim‐3 and Galectin‐9 was also observed (Supplemental Figure S11B‐C). Along these lines, a significant increase in the response and a reduction of apoptosis of CD4+ and CD8+ T cells from COVID‐19 patients was experienced when anti‐Tim‐3 and anti‐Galectin‐9 blocking antibodies were used ex vivo (Figure S11D‐G).

We moved to analyse whether ICs are stronger severity biomarkers than cytokines in COVID‐19. We analysed the levels of 12 cytokines/chemokines in our cohort. Only CCL‐2, CXCL10, IL‐6 and IL‐8 showed significant differences between groups (Supplemental Figure S13). Next, using an area under the ROC curve (Area Under the Curve/Receiver Operating Characteristic [AUC/ROC]) analysis, we compared ability as predictor of the ICs, chemokines and cytokines. We found sCD25 and sTim‐3 exhibited the highest AUCs for both mortality and ICU requirement prediction (Supplemental Figures S14 and S15) of all the molecules studied.

Beyond ICs and cytokines, we studied the predictive potential of 53 variables including epidemiological, physiological and other relevant parameters by AUC/ROC and univariate regression (Table 1). We performed a binary logistic regression model, including the 14 variables with an AUC/ROC > 0.7 and statistical significance in univariate analysis. After 12 steps (Supplemental Table S2), the final model included age, SpO_2_/FiO_2_, D‐Dimer, sCD25 and sCD86 as predictors of mortality (Figure 3A). The score obtained from this model showed an AUC/ROC of 0.9753 for mortality prediction (Figure 3B). The optimal cut‐off calculated by the Youden index showed a sensitivity of 0.93 and a specificity of 1 (Figure 3C). Note this predictive capacity is remarkably higher compared to the performance of all 53 variables alone (none of them had an AUC/ROC > 0.84; Table 1).

**TABLE 1:** Variables analysed as potential biomarkers for mortality

Variable	ROC			Univariate logistic regression	
	AUC	*p*‐value	OR	OR IC 95	*p*‐value
Age*	**0.8019**	**.0005**	1.0793	1.029183–1.131854	**.0017**
Sex (male)	0.6130	.1942	2.526	0.764313–8.35033	.1287
AHT*	**0.7026**	**.0199**	5.5800	1.533836–20.359662	**.0091**
DM	0.5786	.3667	2.2222	0.619494–7.971464	.2205
CVD	0.5604	.4878	1.7949	0.51024–6.313824	.3621
CKD	0.5974	.2631	4.0000	0.910787–17.567221	.0663
OBESITY	0.5552	.5260	0.3462	0.040472–2.960613	.3327
COPD	0.5883	.3103	3.2667	0.776781–13.73761	.1062
Onco	0.5006	.9940	0.9808	0.100886–9.534592	.9866
ImmunoD	0.5013	.9881	0.9792	0.183563–5.22311	.9803
Temperature	0.5812	.3510	1.1719	0.627006–2.190483	.6191
Heart rate	0.5396	.6490	1.0084	0.976758–1.041001	.6081
Resp rate*	**0.7695**	**.0020**	1.1780	1.048269–1.323747	**.0059**
SpO_2_ *	**0.7318**	**.0077**	0.8853	0.797259–0.982972	**.0225**
FiO_2_	0.6506	.0835	1.2276	1.036604–1.453895	**.0175**
SpO_2_/FiO_2_ *	**0.8214**	**.0002**	0.9802	0.968543–0.99202	**.0011**
Lactate*	**0.8364**	**.0001**	9.2471	2.633093–32.474967	**.0005**
Absolute leukocytes	0.5513	.5556	1.0000	0.999919–1.000092	.8960
ANC	0.5987	.2568	1.0002	0.999983–1.000375	.0733
ALC*	**0.8234**	**.0002**	0.9969	0.994924–0.99884	**.0018**
AMC	0.6110	.2020	0.9983	0.99464–1.001913	.3514
N/L ratio*	**0.7974**	**.0006**	1.0658	1.005046–1.13017	**.0333**
Platelets*	**0.7383**	**.0062**	0.9999	0.999985–0.999998	**.0095**
Ferritin	**0.7403**	**.0058**	1.0005	0.999808–1.001173	.1588
D‐Dimer*	**0.7435**	**.0051**	1.0004	1.000033–1.000805	**.0332**
Creatinine	**0.7221**	**.0107**	1.1703	0.793126–1.726702	.4283
AST	0.5117	.8932	1.0037	0.997514–1.009897	.2423
ALT	0.5416	.6330	0.9924	0.974032–1.011074	.4218
LDH	0.6558	.0734	1.0037	0.999925–1.007448	.0548
CRP	0.6981	**.0229**	1.0044	0.998406–1.010426	.1506
PCT	**0.7110**	**.0153**	1.0199	0.984334–1.05684	.2761
qSOFA*	**0.8253**	**.0002**	9.3700	2.7216–32.259104	**.0004**
sCD25*	**0.7708**	**.0019**	1.0005	1.000001–1.001091	**.0497**
sCD86*	**0.7250**	**.0096**	0.9893	0.980009–0.998608	**.0244**
sCD137	0.5266	.7597	1.0011	0.988669–1.013693	.8628
sCTLA‐4	0.5818	.3472	0.1124	0.822844–0.646805	1.0468
Galectin‐9	0.6883	**.0305**	1.0000	0.999998–1.000012	.1552
sLAG‐3	0.6052	.2268	1.0001	0.999987–1.000254	.0759
sPD‐1	0.6110	.2020	1.0211	0.99–1.052975	.1824
sPD‐L1	0.6920	**.0267**	1.0129	0.999984–1.025989	.0503
sTim‐3*	**0.7620**	**.0025**	1.0001	1.000011–1.000101	**.0138**
CCL‐2	0.6792	**.0395**	1.0033	1.000145–1.006449	**.0403**
CXCL10	0.6883	**.0305**	1.0006	0.999967–1.001227	.0633
IFNg	0.5571	.5115	1.0010	0.998441–1.003467	.4582
IL‐1β	0.5227	.7940	1.0389	0.872492–1.236977	.6685
IL‐2	0.5922	.2894	1.1051	0.962296–1.269112	.1569
IL‐4	0.5961	.2695	0.9703	0.921224–1.022008	.2551
IL‐6	**0.7169**	**.0127**	1.0022	0.999664–1.004802	.0885
IL‐8	0.6409	.1055	1.0307	1.004042–1.05814	**.0237**
IL‐10	0.5338	.6981	1.0058	0.9953–1.016485	.2787
IL‐12p70	0.5597	.4925	0.9428	0.860632–1.032824	.2056
IL‐17A	0.5701	.4204	0.9293	0.801122–1.07795	.3327
TNFα	0.567	.442	1.00005	0.913573–1.094726	.9903

The asterisk * labels those variables included in the Wald backward stepwise regression model. In bold are the areas under the ROC curve (AUC) > 0.5 and the *p*‐values < .05.

Abbreviations: AHT, arterial hypertension; DM, history of diabetes mellitus; CVD, history of cardiovascular disease; CKD, history of chronic kidney disease; COPD, history of chronic obstructive pulmonary disease; OncoD, history of oncologic disease; ImmunoD, history of immunologic disease; Resp rate, respiratory rate; SpO_2_, oxygen saturation; FiO_2_, fraction of inspired oxygen; SpO_2_/FiO_2_, peripheral blood oxygen saturation to fraction of inspired oxygen ratio; ANC, absolute neutrophil counts; ALC, absolute lymphocyte counts; AMC, absolute monocyte counts; N/L Ratio, neutrophil to lymphocyte ratio; AST, aspartate transaminase; ALT, alanine transaminase; LDH, lactate dehydrogenase; CRP, c‐reactive protein, PCT, procalcitonin; qSOFA, quick sequential organ failure assessment score.

**FIGURE 3:**
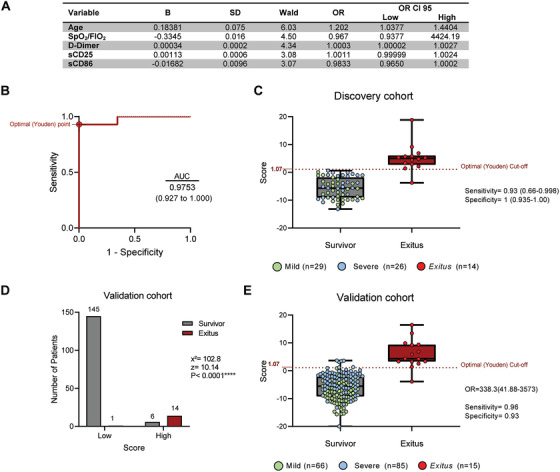
Score from the logistic mathematical model predicted the mortality of patients with COVID‐19 in a validation cohort. (A) Wald backward stepwise regression, including, as variables, age, previous diagnosis of hypertension, respiratory rate, pulse oximetric saturation/fraction of inspired O_2_ ratio (SpO_2_/FIO_2_), lactate, ALC, ratio neutrophil/lymphocyte, platelets, D‐Dimer, qSOFA, sCD25, sCD86 and sTim‐3. The final model after 12 steps is shown. Units: age in years; SpO_2_/FiO_2_ in arbitrary units, D‐Dimer in ng/mL, sCD25 and sCD86 in pg/mL. (B) ROC curve analysis for mortality prediction in discovery cohort (*n* = 69) of the score obtained from the logistic regression model including age, D‐Dimer, sCD86, sCD25 and SpO_2_/FiO_2_ as variables. The optimal Youden point is shown. (C) Estimated score of discovery COVID‐19 patients’ cohort according to their outcome. The dashed line indicates the optimal Youden cut‐off for mortality prediction. Sensitivity and specificity of this cut‐off is shown. (D) Patients from validation cohort (*n* = 166) were classified according to their outcome and the regression model score (low and high subgroups by using the optimal Youden cut‐off). Patient number distribution and chi‐square test statistics are shown. (E) Estimated score of validation COVID‐19 patients’ cohort according to their outcome. The dashed line indicates the optimal Youden cut‐off for mortality prediction. Data represented in box‐and‐whisker plots (min to max). Odds ratio (OR), sensitivity and specificity of this cut‐off are shown. B, B weight coefficient; OR, odds ratio; OR CI 95, 95% confidence interval of odds ratio; SD, standard deviation of B; Wald, Wald statistic X^2^, chi‐square; z, *z*‐statistic.

To corroborate the relevance of this score, we estimated it in a prospective validation independent cohort of COVID‐19 patients (*n* = 166, Supplemental Table S3). We classified those patients into two groups according to the cut‐off estimated in the discovery cohort (Figure 3D). The chi‐squared test showed statistically significant differences in mortality frequency between the groups (χ^2^ = 102.8; *z* = 10.14; Figure 3D), and the score performed the discrimination of survivors and exitus with a specificity of 0.93 and a sensitivity of 0.96 (Figure 3E).

In summary, our data demonstrated IC levels on admission are better mortality predictors than other biomarkers such as cytokines in COVID‐19. Combination of ICs and other easily measurable parameters identifies at ED admission those patients with the worst outcome. In addition, our ex vivo assays suggested the potential of ICs as pharmaceutical targets.

## CONFLICT OF INTEREST

The authors have declared that no conflict of interest exists.

## Supporting information

Supporting Information

Supporting Information

Supporting Information

Supporting Information
