# Supplementary material for: The immune checkpoints storm in COVID‐19: Role as severity markers at emergency department admission
Source: Clin Transl Med. 2021 Oct 18;11(10):e573. doi: 10.1002/ctm2.573 (PMC8521292; doi:10.1002/ctm2.573)
Supplement: Supplementary file 1 — Supporting Information [file CTM2-11-e573-s003.docx]

**SUPPORTING INFORMATION**

***The immune checkpoints storm in COVID-19: role as severity markers at emergency department admission***

José Avendaño-Ortiz, Roberto Lozano-Rodríguez, Alejandro Martín-Quirós, Verónica Terrón, Charbel Maroun-Eid, Karla Montalbán-Hernández, Jaime Valentín Quiroga, Miguel Ángel García-Garrido, Elena Muñoz del Val, Álvaro del Balzo-Castillo, María Peinado, Laura Gómez, Carmen Herrero-Benito, Carolina Rubio, José Carlos Casalvilla-Dueñas, Paloma Gómez-Campelo, Alejandro Pascual-Iglesias, Carlos del Fresno, Luis A. Aguirre and Eduardo López-Collazo

## **LIST OF ABBREVIATIONS:**

ALC, absolute lymphocyte count; ANOVA, analysis of variance; AUC, area under a curve; CFSE, carboxyfluorescein succinimidyl ester; CI, confidence interval; COVID-19, coronavirus disease 19; ED, emergency department; EDTA, ethylenediaminetetraacetic acid; ELISA, enzyme-linked immunosorbent assay; FBS, fetal bovine serum; FiO2, fraction of inspired oxygen; HIV, human immunodeficiency virus; HV, healthy volunteer; IC, immune checkpoint; ICU, intensive care unit; IgG, immunoglobulin G; IgM, immunoglobulin M; IL, i nterleukin; KW, Kruskal-Wallis statistic; OR, odds ratio; PBMC, peripheral blood mononuclear cell; PCA, principal component analysis; PWD, pokeweed mitogen; RPMI, Roswell park memorial institute; ROC, receiver operating characteristic; RT-qPCR, reverse transcriptase quantitative polymerase chain reaction; SARS-CoV-2, severe acute respiratory syndrome coronavirus 2; SEM, standard error of the mean; SaO_2_, saturation of oxygen (arterial blood); SpO_2_/FiO_2_, peripheral blood oxygen saturation to fraction of inspired oxygen ratio; q-SOFA, quick sepsis related organ failure assessment.

**SUPPORTING MATERIAL AND METHODS**

**Patient recruitment and sample collection**

We designed a prospective observational analysis (STROBE guidelines checklist in online supplemental material) with two cohorts of patients with COVID-19 (discovery cohort, n=69, and validation cohort, n=166) and healthy volunteers (HV, n=15). All HVs had no history of any significant systemic diseases or malignancy, were asymptomatic for more than 14 days, and were negative for both SARS-CoV-2 RT-PCR test and negative to antiSARS-CoV-2 -IgM and IgG. Due to the descriptive characteristics of our study, there was no predefined sample size. For both discovery and validation cohorts, patients were included when they met the diagnostic criteria for COVID-19 and were positive for SARS-CoV-2 by RT-qPCR or antigen test (Roche) from nasopharyngeal swabs. Patients were consecutively recruited from the ED solely when the clinical investigation team was available, independently of the patients’ clinical status and prior to their hospitalization in La Paz University Hospital in Madrid (Spain). Patients receiving immunosuppressants (i.e.: chemotherapy, chronic high doses of corticosteroids) and those with immunodeficiency (primary or acquired) were excluded from the study. For each patient we took a sample on admission and before any treatment was performed. In addition, we collected longitudinal samples every 2–4 days until discharge or *exitus*. All participants signed an informed consent and data were anonymized before study inclusion, and their details are summarized in **Supplementary Table 1**.

For the discovery cohort, patients were enrolled from April 28 to September 21, 2020. The patients who died during the first 28-day of hospitalization were defined as *exitus* (n=14) and the patients who recovered during hospitalization were defined as survivors/discharged (n=55). COVID-19 death was defined according to code U07.1 from “International guidelines for certification and classification (coding) of COVID-19 as cause of death” 20 April 2020 version from WHO. Patients identified as survivors were sub-divided into two groups according to their maximum health care requirement during their stay: no O_2_ requirement (mild, n=29) and hospitalized with O_2_ supply (severe, n=26). For the longitudinal analysis, samples were collected each 2–4 days until discharge or *exitus*. There was no loss of follow-up or readmissions in other hospitals.

Patients receiving immunosuppressants (i.e.: chemotherapy, chronic high doses of corticosteroids) and those with impaired immunity (primary or acquired) were excluded from the study.

Prospectively-collected plasma samples from COVID-19 patients (n=166) recruited from January 11 to March 26, 2021 in La Paz University Hospital were used as a validation independent cohort; demographics and baseline characteristics of these patients are summarized in **Supplementary** **Table 3**.

**Peripheral blood mononuclear cell isolation**

For the culture of peripheral blood mononuclear cells (PBMCs) from patients with COVID-19 under the biosafety level 3 (BSL3), EDTA anticoagulant venous blood samples were sent to the GlaxoSmithKline laboratory in Tres Cantos, Spain. There, PBMCs were isolated from venous blood using Ficoll-Plus (GE Healthcare Bio-Sciences) solution according to standard density gradient centrifugation methods.

**Plasma collection**

Plasma samples from HVs and patients were obtained from EDTA anticoagulant venous blood using Ficoll-Plus (GE Healthcare Bio-Sciences) according to the standard density gradient centrifugation method. Samples were aliquoted and stored at −80 °C until their analysis.

**Soluble immune checkpoints and cytokine quantification**

The reserved plasma samples from HVs and patients with COVID-19 stored at −80 °C were thawed and centrifuged at 1000 relative centrifugal force for 20 min to remove particulates prior to use. The concentration measurements of cytokines and soluble ICs in plasma samples were performed by the bead-based multiplex assays, LEGENDplex Human Inflammation Panel 1 (12-plex: IL-1β, IL-2, IL-4, IFN-γ, TNF-α, MCP-1 (CCL2), CXCL10, IL-6, IL-8 (CXCL8), IL-10, IL-12p70 and IL-17A) (BioLegend) and LEGENDplex HU Immune Checkpoint Panel 1 (9-plex: sCD25, sCD137, sCD86, sCTLA-4, sPD-L1, sPD-1, sTim-3, sLAG-3 and Galectin-9) (BioLegend), respectively, according to the manufacturer’s instructions. Briefly, no diluted patients’ plasma was incubated for 2 hours with fluorescent capture beads for 2 hours. After that, beads were washed and incubated with biotinylated secondary detection antibodies for 1 hour, after that they were incubated with Streptavidin-Phycoerythrin for detecting biotinylated antibodies. After that, beads were washed and acquired on a FACSCalibur flow cytometer (BD Biosciences). Calibration curves with known concentrations of each analyte were prepared in parallel to samples every day of analysis. Data were analyzed using LEGENDplex (BioLegend) v.8 software.

**Antibodies and immunophenotyping by flow cytometry**

For the analysis of the blood cell subsets and ICs by surface marker expression, an aliquot (2 mL) of heparinized blood from HVs and patients with COVID-19 was incubated with 20 mL of 1X Pharm Lyse Buffer (BD Biosciences) for 5 minutes at room temperature (RT) in a rocker to lyse red blood cells. Next, it was washed twice in phosphate-buffered saline, and dead cells were stained with LIVE/DEAD Fixable Blue Dead Cell Stain Kit (Invitrogen) for 15 minutes. True-Stain Monocyte Blocker (BioLegend) reagent was added prior to the label protocol to block the nonspecific binding of some fluorochromes to monocytes. Finally, resulting cells were labeled with an 8-color antibody cocktail for 25 min at RT in the dark with fluorochrome-conjugated monoclonal antibodies. The list of the fluorochrome-conjugated monoclonal antibodies used is shown in **Supplementary Table 4**. Labeled cells were acquired on a Cytek Aurora Spectral Cytometer (Cytek Biosciences). Data were analyzed using FlowJo (TreeStar) v10.6.2 software. The gating strategy followed is shown in **Supplementary Figure 4.**

**Lymphocyte proliferation assay**

Peripheral blood mononuclear cells (PBMCs) were isolated using Ficoll-Plus (GE Healthcare Bio-Sciences. CFSE-labelled PBMCs (2x10^5^ cells per well) were stimulated or not with pokeweed mitogen (PWD, 2.5 μg/mL purchased from ThermoFisher Scientific) for 5 days in RPMI 1640 medium, and supplemented with 10% of FBS and 1% penicillin and streptomycin mix. After the 5 days, PBMCs were labelled with CD4-peridinin-chlorophyll-A protein and CD8-allophycocyanin (both from ImmunoStep) for 25 min and fixed with 4% paraformaldehyde for 10 min at RT. Cells were acquired in a FACSCalibur (BD Bio-Sciences) flow cytometer and data were analyzed with FlowJo (TreeStar) v10.6.2 software.

**Apoptosis assay**

PBMCs (2x10^5^ per well) from patients with COVID-19 were cultured with or without mouse anti-human Galectin-9, rat anti-human Tim-3, chimeric human/murine anti-human PD-1 (Pembrolizumab), and chimeric human/murine anti-human CD25 (Basiliximab) antibodies for 72 hours; 10 μg/mL of each antibody was used. Next, PBMCs were stained with both propidium iodide and Annexin-V-FITC using the Apoptosis Detection Kit of ImmunoStep to measure apoptosis by flow cytometry in a FACSCalibur flow cytometer. Data were analyzed with FlowJo (TreeStar) v.10.6.2 software.

**Statistics**

Data are presented as numbers, percentages, means, and standard deviations. Differences between groups were evaluated with the use of a chi-squared test for categorical variables; Student’s t-test for comparison of quantitative variables between two groups, and Kruskal-Wallis for comparisons of quantitative variables between multiple groups. Correlations between quantitative variables were evaluated by Spearman’s analysis. Receiver operating characteristic (ROC) curve analysis was used to determine whether the cytokines or soluble ICs in plasma levels could be used as a predictor of mortality and/or illness. All optimal cut-off values were estimated by the Youden index. P-values of less than 0.05 were considered to indicate statistical significance. All P-values are 2-sided, and 95% confidence intervals (95% CI) are also presented. Statistical analyses were conducted using Prism 8.0 (GraphPad) and SPSS version 23 (IBM) software.

A logistic regression model for mortality prediction was performed by Wald backward stepwise regression. Briefly, the Wald automatic stepwise selection method includes all the independent variables and gradually removes and reintroduces variables at each step until only the explanatory one remains (Qinggang W. et al. *Communications in Statistics-Simulation and Computation* (2007), 37:1, 62-72, DOI: 10.1080/03610910701723625). We included the 14 variables with AUC/ROC >0.7 and statistical significance in univariate regression (**Table 1**): Age, previous diagnosis of hypertension, respiratory rate, SpO_2_, SpO_2_/FiO_2_ ratio, lactate, ALC, neutrophils /lymphocytes ratio, platelets, D-Dimer, q-SOFA, sCD25, sTim-3 and sCD86. The final model after twelve steps (**Supplementary Table 2**) score can be calculated with the following formula: Score = 0.18381 x Age - 0.3345 x SpO_2_/FiO_2_ + 0.00034 x D-Dimer + 0.00113 x sCD25 - 0.01682 x sCD86 (**Figure 3A**).

Radar plot diagram of IC levels in HVs and COVID-19 patients’ plasma on admission was performed using the average of Z-score by VisualParadigm online tool. Heatmap analysis of individual Z-scores for each IC level in HVs and COVID-19 patients’ plasma was performed according to the severity of the groups using GraphPad v. 8.0 software. Principal Components Analysis (PCA), including the variables sCD25, sCD86, sPD-L1, sTim-3 and Galectin-9, was calculated using the unsupervised function princomp of R language. The calculation was done by a singular value decomposition of the (centered and scaled) data matrix. The print method for these objects and plot was made by the packages R ggplot and ggbiplot.

**Ethics approval**

Informed consent was obtained from all participants in accordance with the hospital’s ethical standards and following the ethical guidelines of the 1975 Declaration of Helsinki. The study was authorized by the La Paz University Hospital Research Ethics Committee (PI-4100).

**Study limitation**

Our data indicate that ICs are robust biomarkers for COVID-19 severity. Nevertheless, the sample size is a limitation of our study. A larger multi-center longitudinal study would be recommended.
