# Supplementary material for: The immune checkpoints storm in COVID‐19: Role as severity markers at emergency department admission
Source: Clin Transl Med. 2021 Oct 18;11(10):e573. doi: 10.1002/ctm2.573 (PMC8521292; doi:10.1002/ctm2.573)
Supplement: Supplementary file 2 — Supporting Information [file CTM2-11-e573-s004.docx]

**SUPPORTING INFORMATION: SUPPLEMENTARY FIGURES**

***The immune checkpoints storm in COVID-19: role as severity markers at emergency department admission***

José Avendaño-Ortiz, Roberto Lozano-Rodríguez, Alejandro Martín-Quirós, Verónica Terrón, Charbel Maroun-Eid, Karla Montalbán-Hernández, Jaime Valentín Quiroga, Miguel Ángel García-Garrido, Elena Muñoz del Val, Álvaro del Balzo-Castillo, María Peinado, Laura Gómez, Carmen Herrero-Benito, Carolina Rubio, José Carlos Casalvilla-Dueñas, Paloma Gómez-Campelo, Alejandro Pascual-Iglesias, Carlos del Fresno, Luis A. Aguirre and Eduardo López-Collazo

**SUPPLEMENTARY FIGURES**

**Supplementary Figure 1**

**
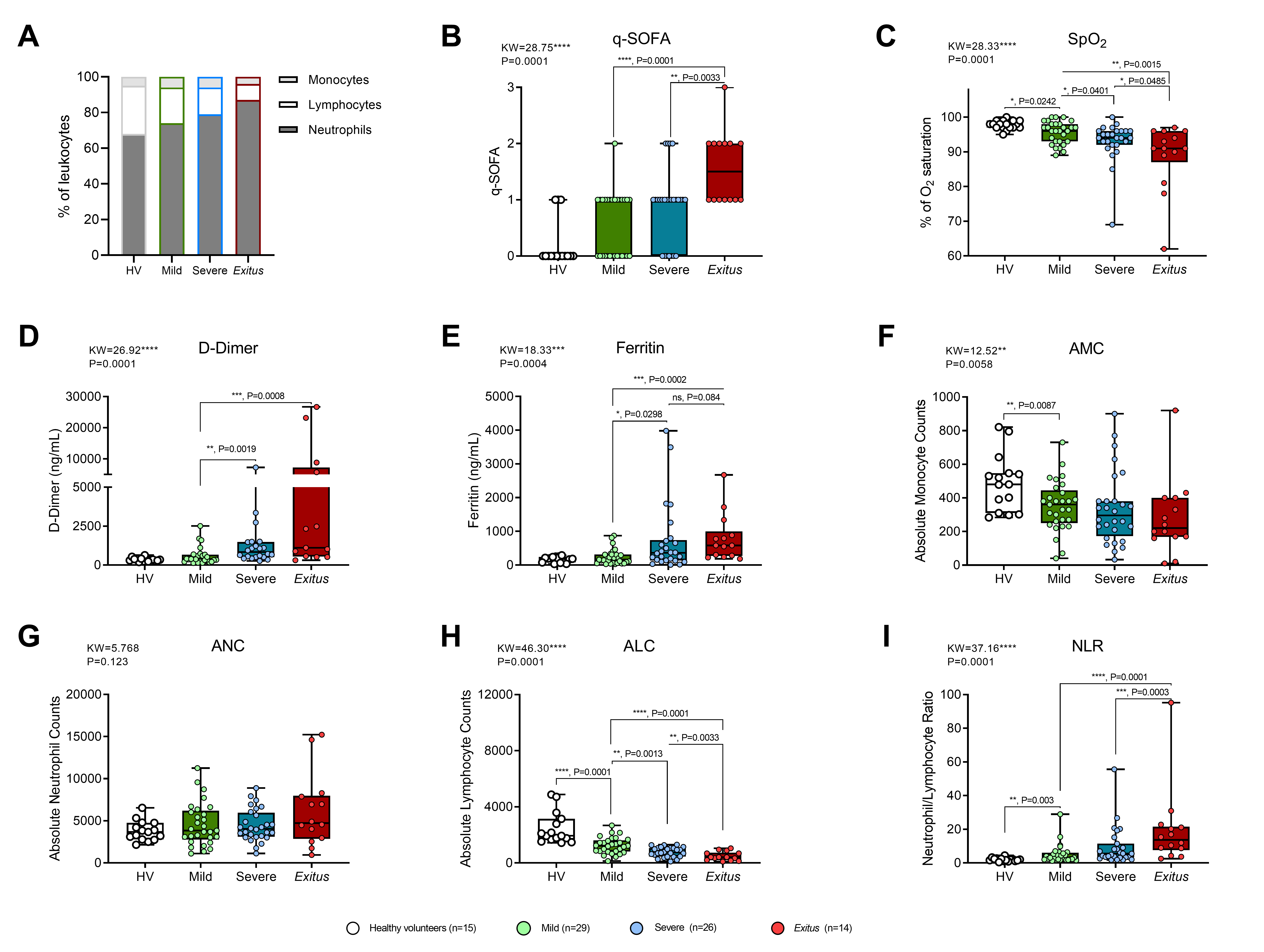
Supplementary Figure 1. COVID-19 illness evolution based on blood cellular and biochemical parameters on admission.** (**A**) Distribution of the main blood cells (monocytes, lymphocytes, and neutrophils) in HVs (n=15) and patients with COVID-19 on admission according to the severity group: Mild (n=29); Severe (n=26); and *exitus* (n=14) are shown. Severity-associated changes in clinical parameters in patients with COVID-19 on admission compared with HVs: q-SOFA (quick Sequential Organ Failure Assessment) (**B**); SpO_2_ (Saturation of O_2_) (**C**); D-Dimer (**D**); Ferritin (**E**); AMC (absolute monocyte count, counts/mm^3^) (**F**); ANC (absolute neutrophil count, counts/mm^3^) (**G**); ALC (absolute lymphocyte count, counts/mm^3^) (**H**); and NLR (neutrophils/lymphocytes ratio) (**I**) are shown. Data were analyzed by Kruskal-Wallis and Mann-Whitney U tests. Data represented in box-and-whisker plots (min to max). *, P < 0.05; **, P < 0.01; ***, P < 0.001; ****, P < 0.0001; K-W, Kruskal-Wallis-statistic.

**Supplementary Figure 2**

**
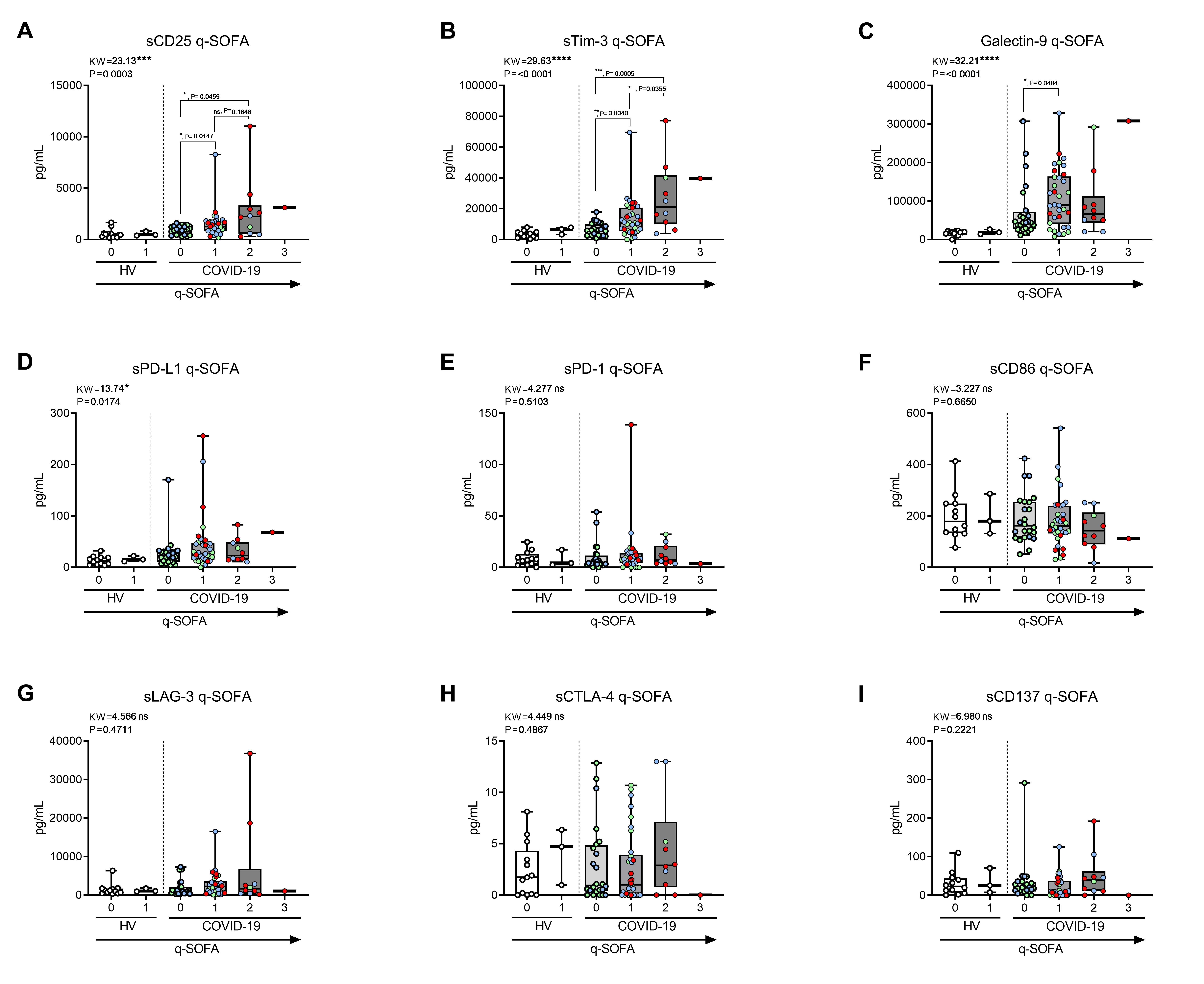
**

**Supplementary Figure 2. Plasma immune checkpoint levels from patients with COVID-19 according to the qSOFA score.** Patients with COVID-19 were classified according to their qSOFA score on admission: 0 (n=25), 1 (n=33), 2 (n=10), and 3 (n=1). Quantification of plasma soluble immune checkpoints according to qSOFA score on admission (0, n=25; 1, n=33; 2, n=10 and 3, n=1) compared with HVs: sCD25 (**A**), sTim-3 (**B**), Galectin-9 (**C**), sPD-L1 (**D**), sPD-1 (**E**), sCD86 (**F**), sLAG-3 (**G**), sCTLA-4 (**H**), and sCD137 (**I**). Data are pg/mL concentrations and were analyzed by Kruskal-Wallis and Mann–Whitney U tests. Data represented in box-and-whisker plots (min to max). *, P < 0.05; ***, P < 0.001; ****, P < 0.0001; KW, Kruskal-Wallis statistic (flora, Mild; orchid, Severe; red, Exitus).

**Supplementary Figure 3**

**
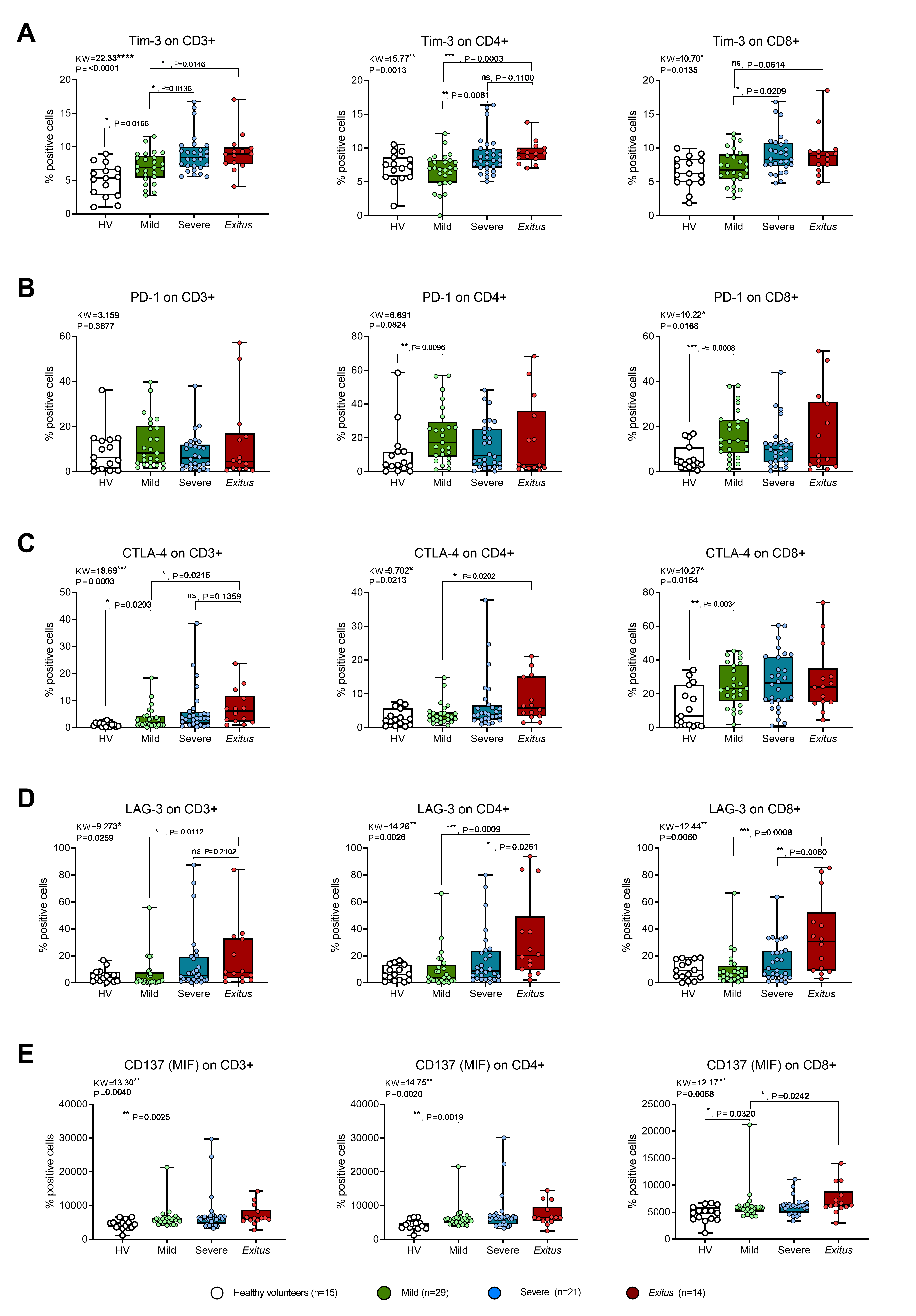
**

**Supplementary Figure 3. Cell surface immune checkpoint expression in patients with COVID-19 on admission according to their outcome.** Percentages of Tim-3 (**A**), PD-1 (**B**), CTLA-4 (**C**), and LAG-3 (**D**) positive cells in HVs and patients with COVID-19 on admission according to the severity group: Mild (n=29); Severe (n=26); and *exitus* (n=14) in CD3^+^ (left panels), CD4^+^ (central panels), and CD8^+^ (right panels) cells are shown. (**E**) Mean fluorescence intensity (MFI) of CD137 in HVs and patients with COVID-19 on admission according to their outcome in CD3^+^ (left panel), CD4^+^ (central panel), and CD8^+^ (right panel) cells are shown. Data are pg/mL concentrations and were analyzed by Kruskal-Wallis and Mann-Whitney U tests. Data represented in box-and-whisker plots (min to max). *, P < 0.05; **, P < 0.01; ***, P < 0.001; ****, P < 0.0001; K-W, Kruskal-Wallis-statistic

**Supplementary Figure 4**


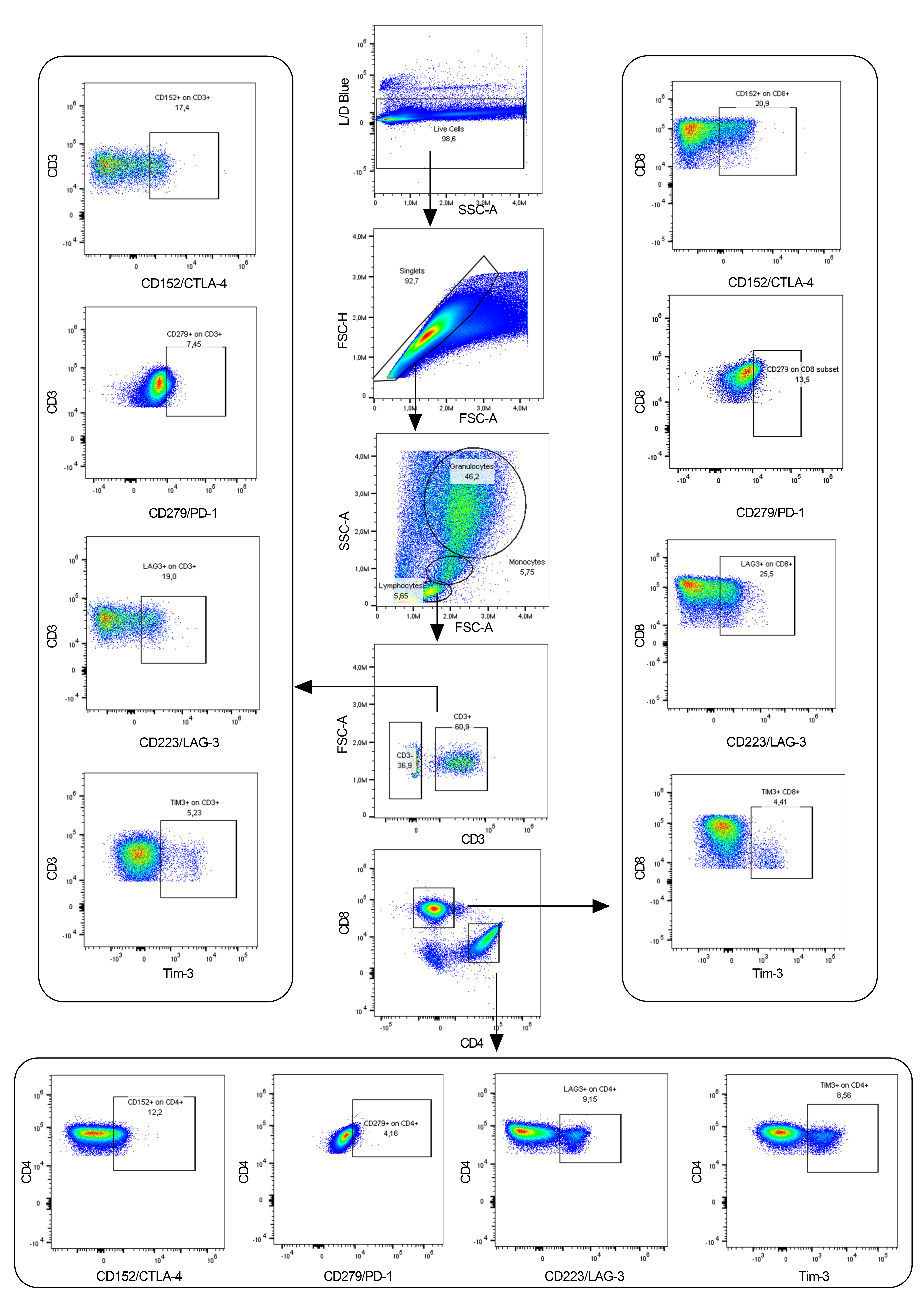


**Supplementary Figure 4. Flow cytometry-gating strategy for membrane immune checkpoint expression in a representative patient with COVID-19 on admission.**

**Supplementary Figure 5**

**
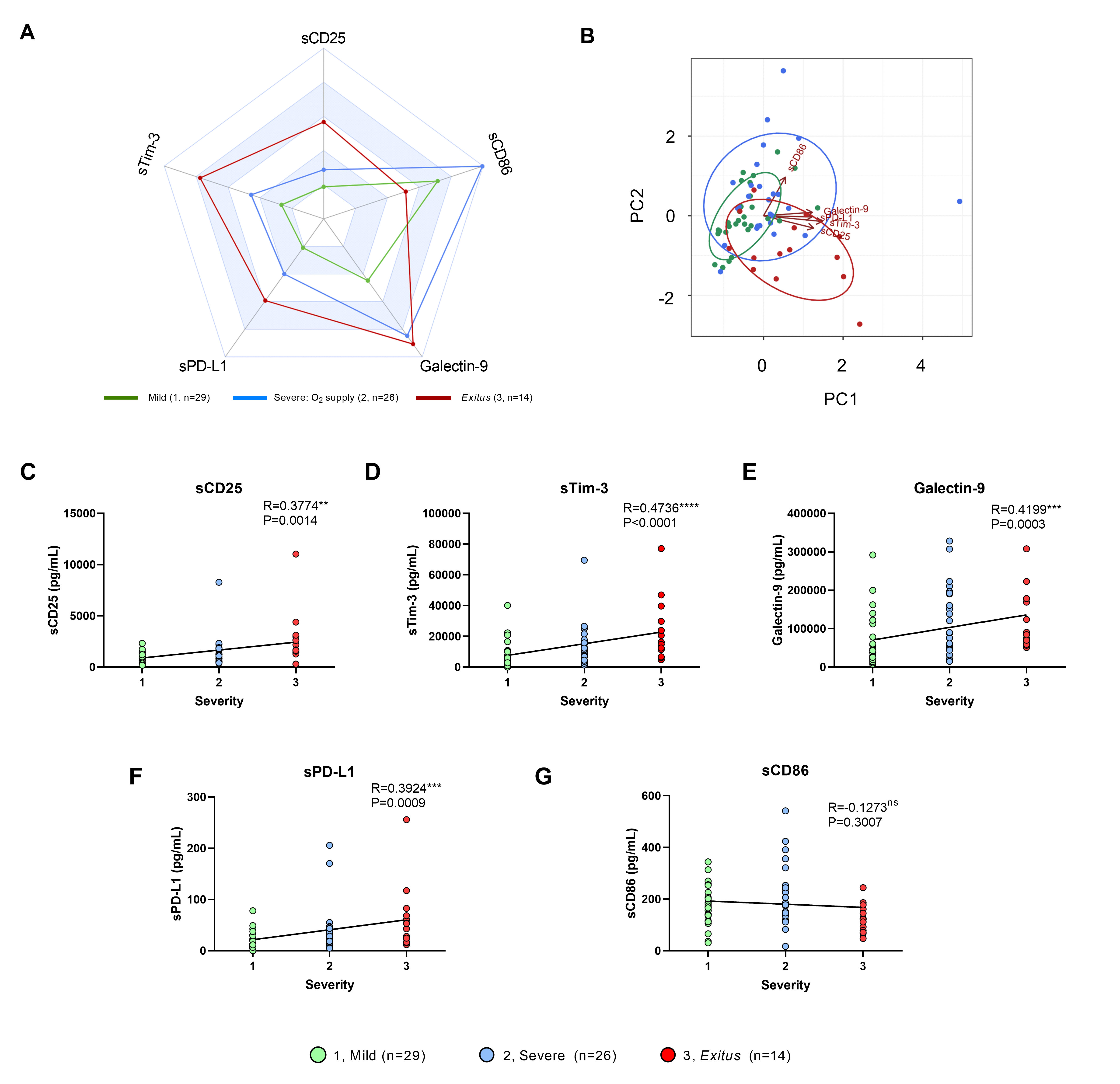
**

**Supplementary Figure 5. Disparate concentration of plasma immune checkpoints differentiates patients with COVID-19 on admission according to their severity.** (**A**) Radar plot of median plasma immune checkpoint level Z-scores (sCD25, sCD86, sPD-L1, sTim-3 and Galectin-9) on admission in patients with COVID-19 grouped by severity/requirement. (**B**) Principal component analysis (PCA) including the plasmatic immune checkpoint levels (sCD25, sCD86, sPD-L1, sTim-3 and Galectin-9) in COVID-19 patients on admission. Colored line ellipses represent 95% confidence intervals around the centroid of each severity/requirement patient subgroups. Spearman correlations of the patient severity (1, Mild; 2, Severe; 3, *Exitus*) and plasma sCD25 (**C**), sTim-3 (**D**), Galectin-9 (**E**), sPD-L1 (**F**), and sCD86 (**G**) concentrations in COVID-19 patients’ plasma on admission are shown (right panels). Data are pg/mL concentrations and correlations were analyzed by Spearman’s test; R, Spearman’s rank correlation coefficient; **P < 0.01; ***P < 0.001; **** P < 0.0001 in Spearman correlation test.

**Supplementary Figure 6**

**
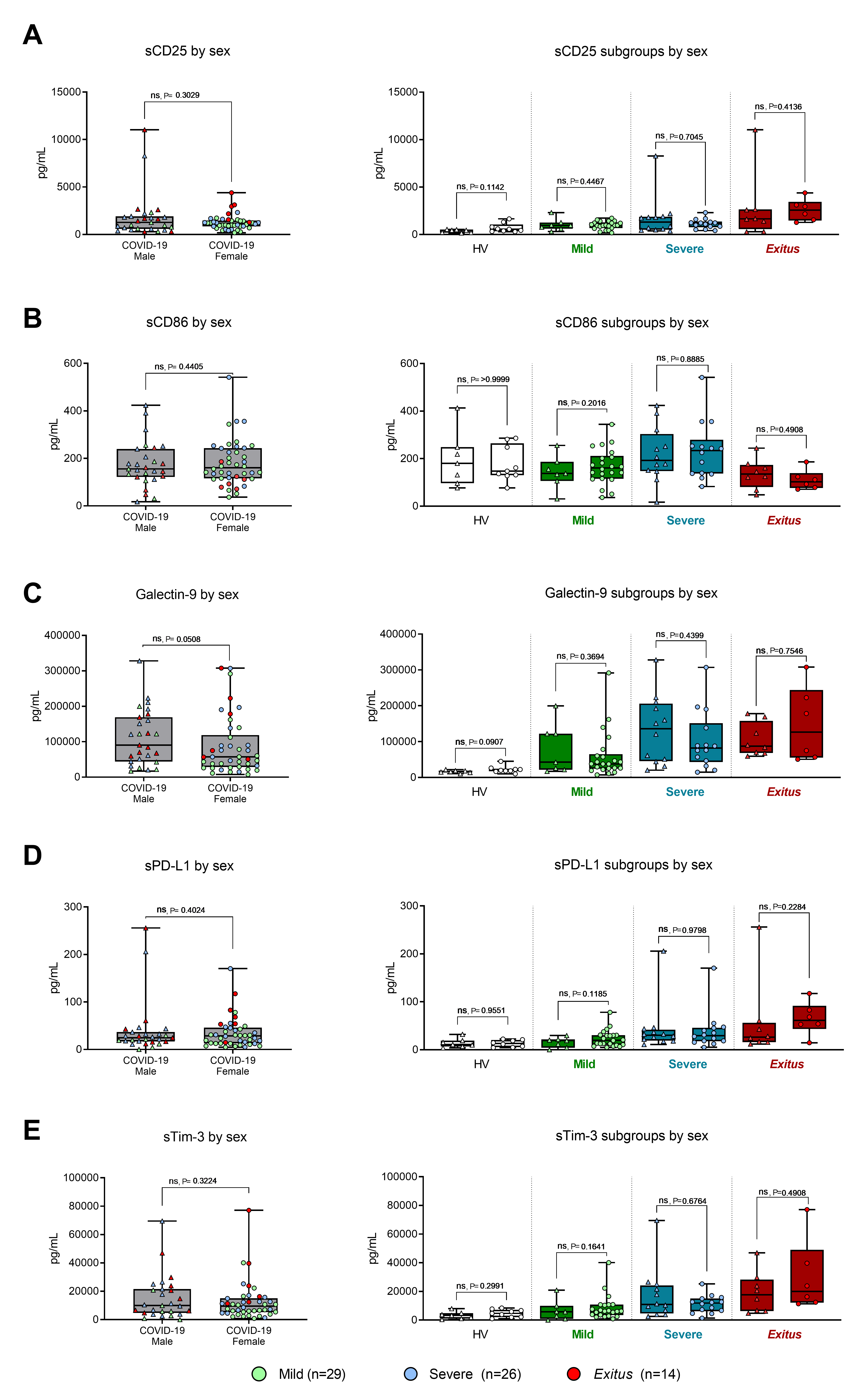
**

**Supplementary Figure 6. Plasma immune checkpoint levels from patients with COVID-19 according to their sex.** Healthy volunteers (HV) and patients with COVID-19 were classified according to their gender: male, n=27; female, n=42 (left panels), and severity (right panels). Plasma levels of sCD25 (**A**), sCD86 (**B**), Galectin-9 (**C**), sPD-L1 (**D**), and sTim-3 (**E**) are shown. Data represented in box-and-whisker plots (min to max). Data are pg/mL concentrations and were analyzed by Mann–Whitney U tests. ns, not significant.

**Supplementary Figure 7**

**
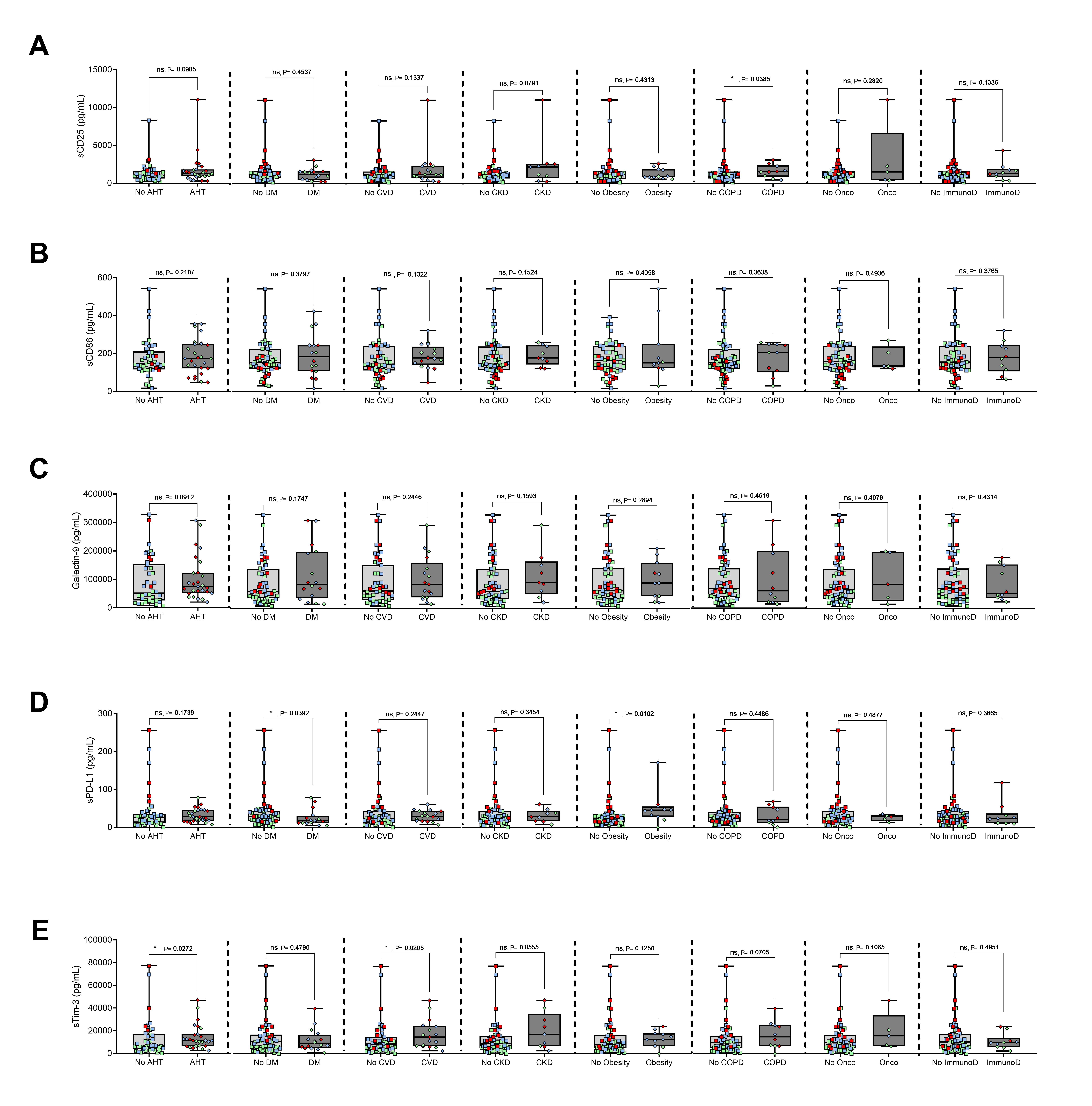
**

**Supplementary Figure 7. Plasma immune checkpoint levels in plasma from patients with COVID-19 according to their comorbidities.** Patients with COVID-19 were classified according to their comorbidities (n of each is shown in **Table 1**). Plasma levels of sCD25 (**A**), sCD86 (**B**), Galectin-9 (**C**), sPD-L1 (**D**), and sTim-3 (**E**) are shown. Data are pg/mL concentrations and were analyzed by Mann–Whitney U tests. Data represented in box-and-whisker plots (min to max). AHT, arterial hypertension; DM, history of diabetes mellitus; CVD, history of cardiovascular disease; CKD, history of chronic kidney disease; COPD, history of chronic obstructive pulmonary disease; Onco, history of oncologic disease; ImmunoD, history of immunologic disease; *, P < 0.05; ns, not significant (flora, Mild; orchid, Severe; red, Exitus).

**Supplementary Figure 8**

**
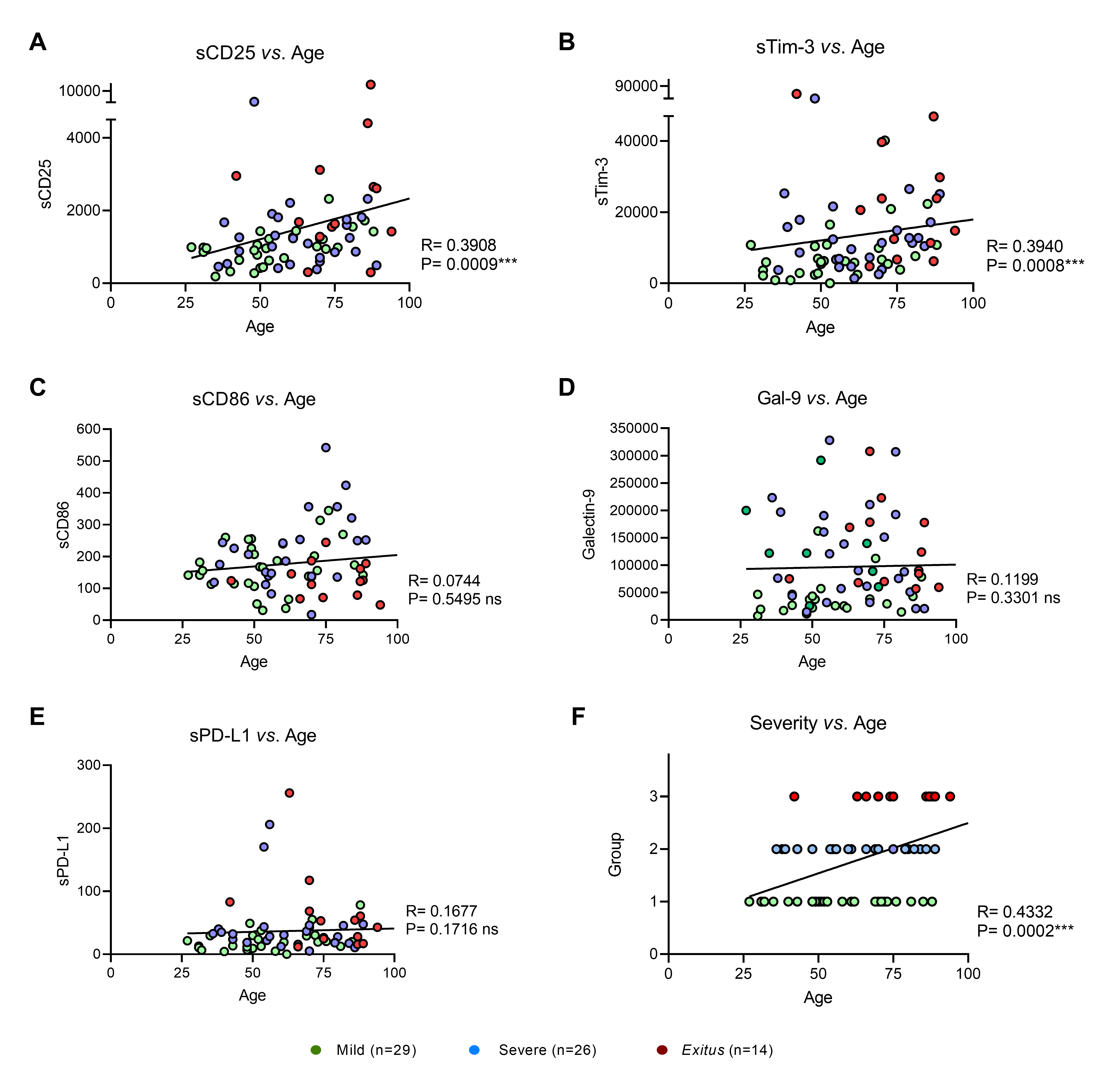
**

**Supplementary Figure 8. Age of patients with COVID-19 positively correlates with severity and plasma sCD25 and sTim-3 levels on admission. (A-E)** Correlations of plasma levels of sCD25 (**A**), sTim-3 (**B**), sCD86 (**C**), Galectin-9 (**D**), and sPD-L1 (**E**) with age, on admission. (**F**) Correlation of severity with age in patients with COVID-19 is shown. Data were analyzed by the Spearman correlation test. R, Spearman’s rank correlation coefficient. ***, P < 0.001; ns, not significant (flora, Mild; orchid, Severe; red, Exitus).

**Supplementary Figure 9**

**
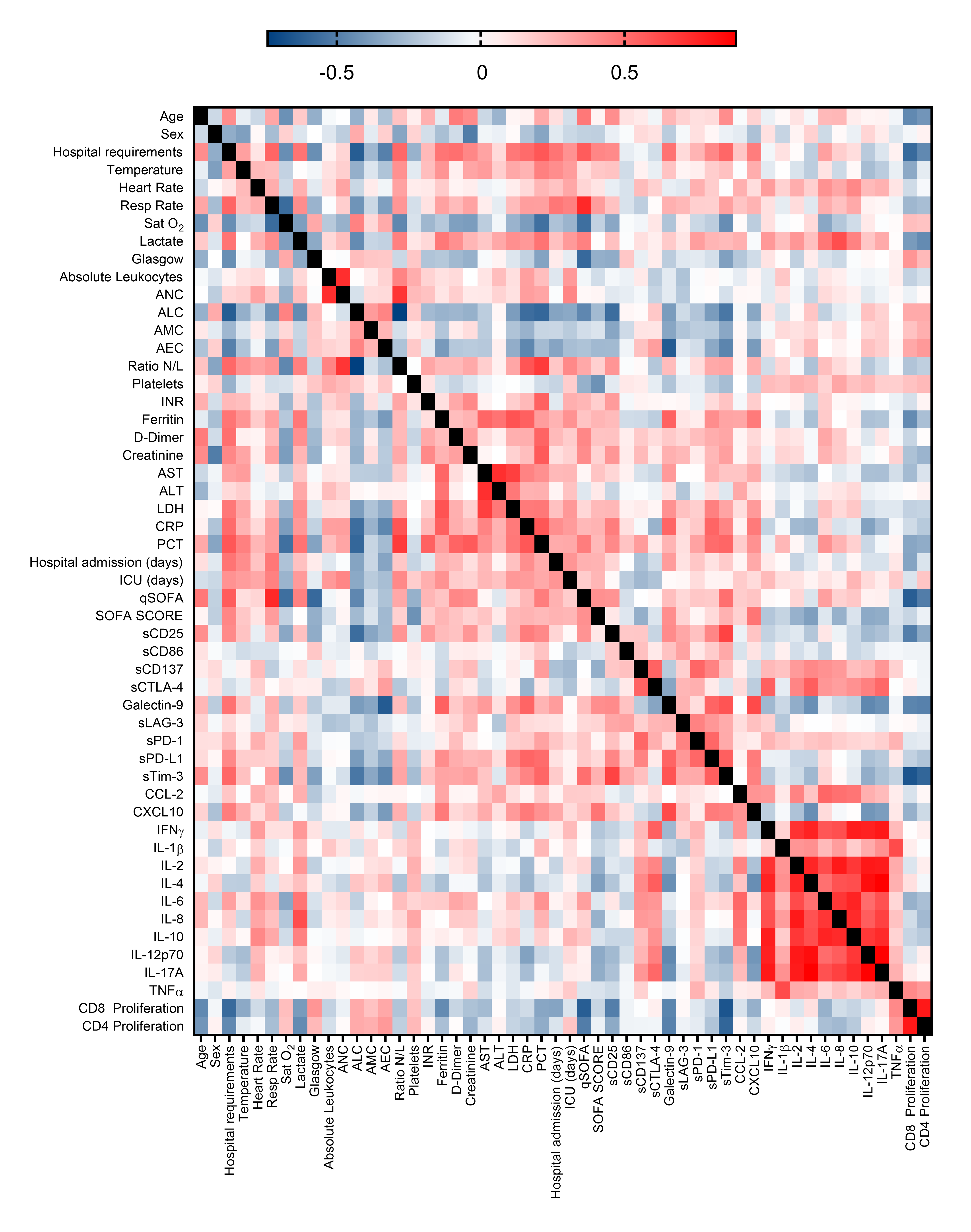
**

**Supplementary Figure 9. Correlation matrix of several clinical parameters, plasma immune checkpoint and cytokine**  **levels of patients with COVID-19 on admission.** Data are Spearman’s rank in all correlations.

**Supplementary Figure 10**

**
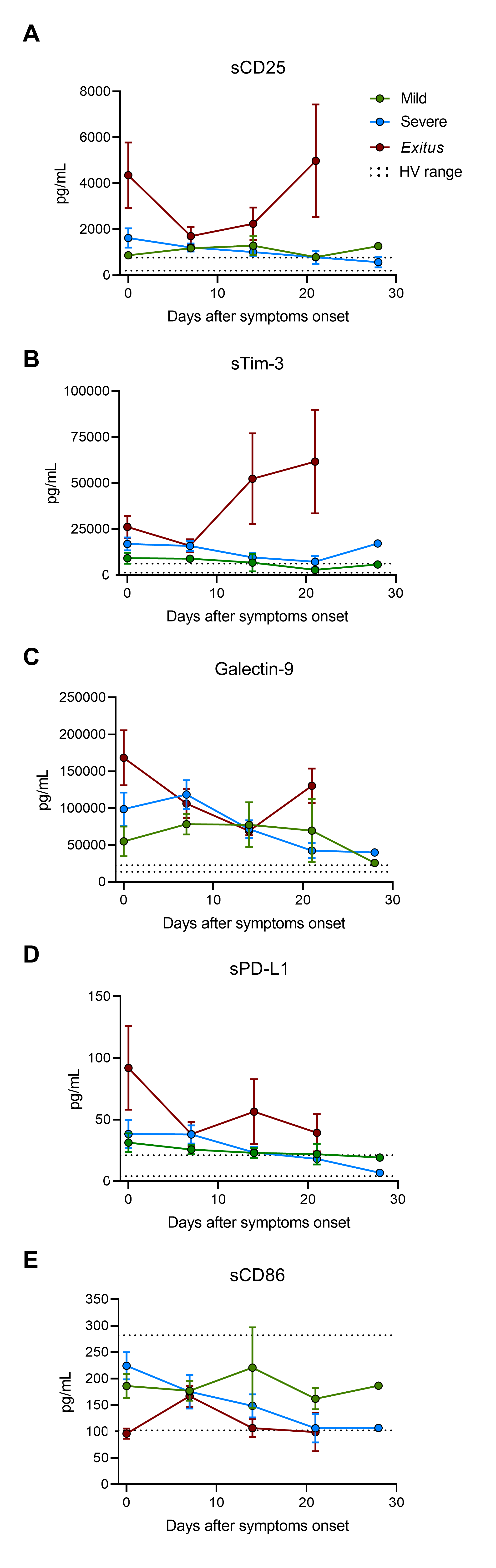
**

**Supplementary Figure 10. Longitudinal analysis of plasma immune checkpoint**  **levels of patients with COVID-19 according to their evolution and oxygen requirements from days after onset of symptoms** **.** Longitudinal concentrations of plasma sCD25 (**A**), sTim-3 (**B**), Galectin-9 (**C**), sPD-L1 (**D**), and sCD86 (**E**) in patients with COVID-19 according to the severity groups from days after onset of symptoms are shown. Data represents mean ± SEM.

**Supplementary Figure 11**

**
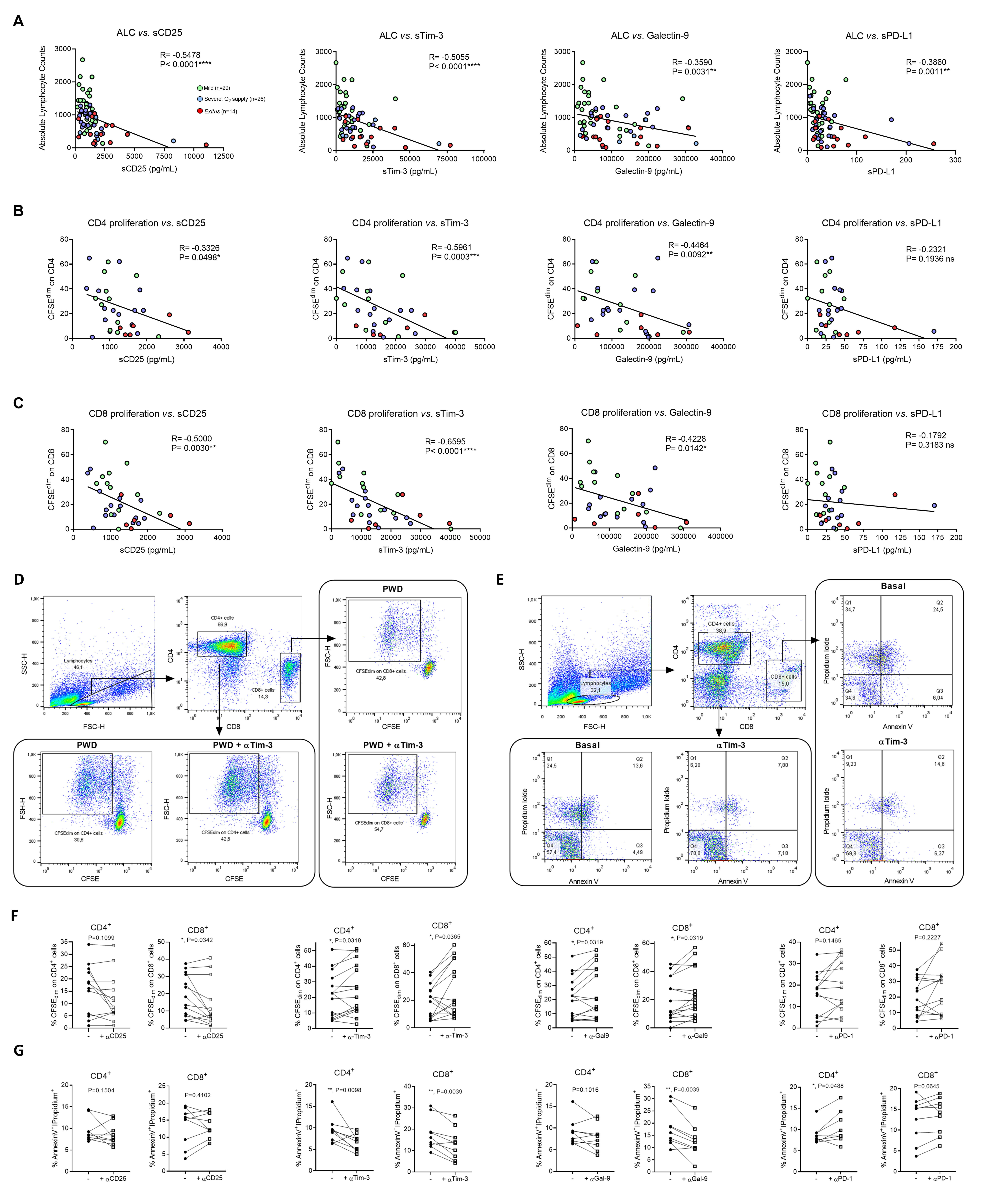
**

**Supplementary Figure 11. Role of sCD25, Galectin-9, sTim-3 and sPD-L1 in the proliferation and apoptosis rate of T cells from COVID-19 patients.** (**A**) Correlations of sCD25 (left panel), sTim-3 (central left panel), Galectin-9 (central right panel), and sPD-L1 (right panel) with absolute lymphocyte counts per mm^3^ of blood (ALCs) are shown. (**B**) Correlations of sCD25 (left panel), sTim-3 (central left panel), Galectin-9 (central right panel), and sPD-L1 (right panel) with proliferation levels of pokeweed (PWD)-stimulated CD4^+^ cells for 5 days are shown. (**C**) Correlations of sCD25 (left panel), sTim-3 (central left panel), Galectin-9 (central right panel), and sPDL1 (right panel) with proliferation levels of PWD-stimulated CD8^+^ cells for 5 days are shown. Representative gating strategy to analyze T cell proliferation (**D**) and apoptosis rate (**E**) in COVID-19 patients treated with blocking antibodies against CD25, Tim-3, Galectin-9 and PD-1. (**F**) Proliferation levels of PWD-stimulated CD4^+^ (left) and CD8^+^ (right) cells of patients with COVID-19 in presence or not of a blocking antibody against CD25 (left panel), Tim-3 (central left panel), Galectin-9 (central right panel) and PD-1 (right panel) for 5 days are shown. (**G**) Percentages of dead cells (AnnexinV^+^/PI^+^ cells) of CD4^+^ (left) and CD8^+^ (right) cells of patients with COVID-19 treated or not with anti-CD25 (left panel), anti-Tim-3 (central left panel), anti-Gal-9 (central right panel) or anti-PD-1 (right panel) for 72 hours are shown. All antibodies were used at a final concentration of 10 μg/mL. Correlations were analyzed by the Spearman correlation test. R, Spearman’s rank correlation coefficient; * P < 0.05; **P < 0.01; ***P < 0.001; **** P < 0.0001. Antibody effects in T cell proliferation and apoptosis rate were analyzed by Wilcoxon signed-rank paired test.

**Supplementary Figure 12**

**
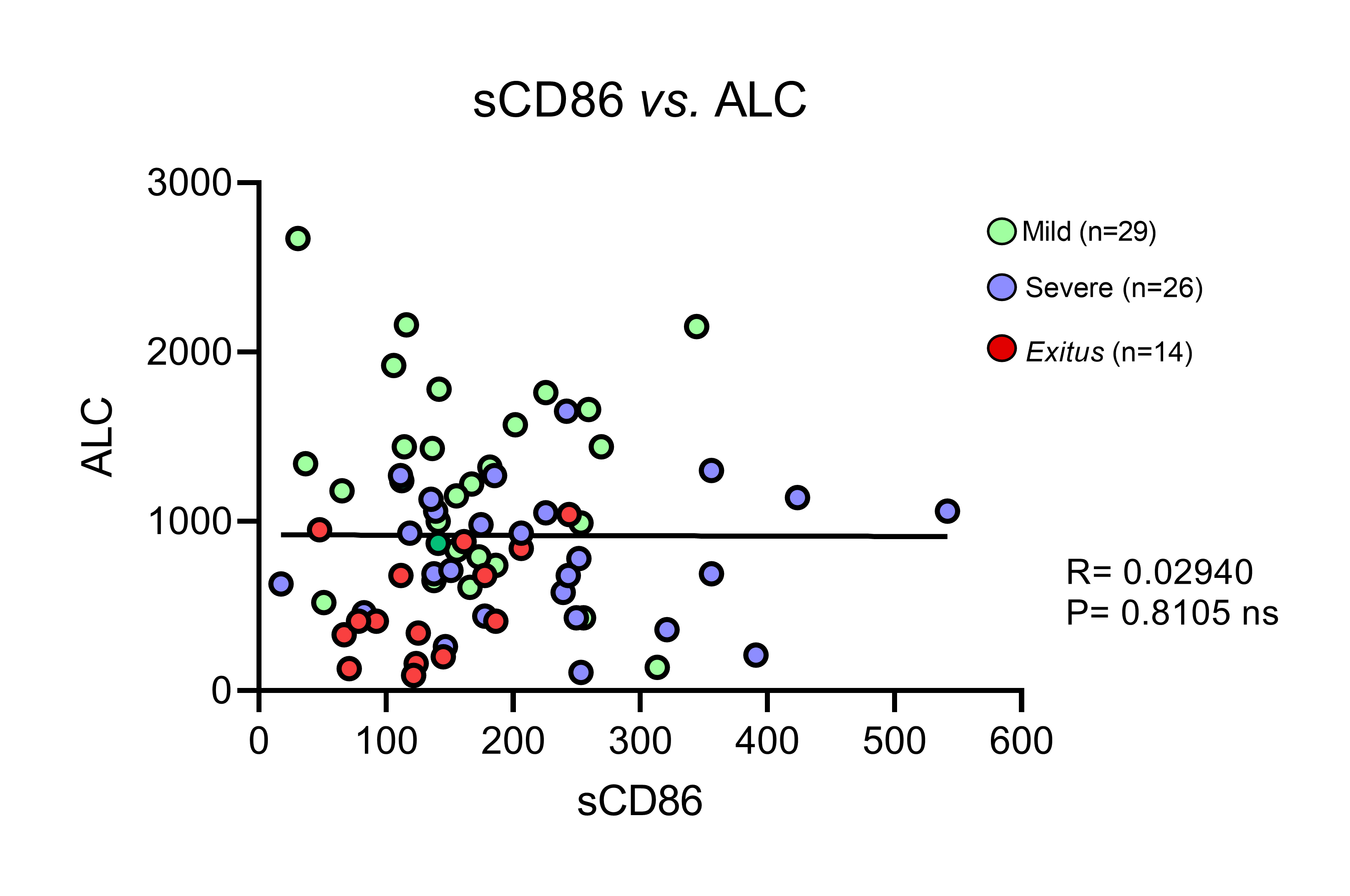
**

**Supplementary Figure 12. Correlation of plasma sCD86 levels of patients with COVID-19 with absolute lymphocyte counts (ALCs) on admission.** Data are pg/mL and cells per mm^3^ respectively and were analyzed by Spearman correlation test. Data are R, Spearman’s rank correlation coefficient ns, not significant.

**Supplementary Figure 13**

**
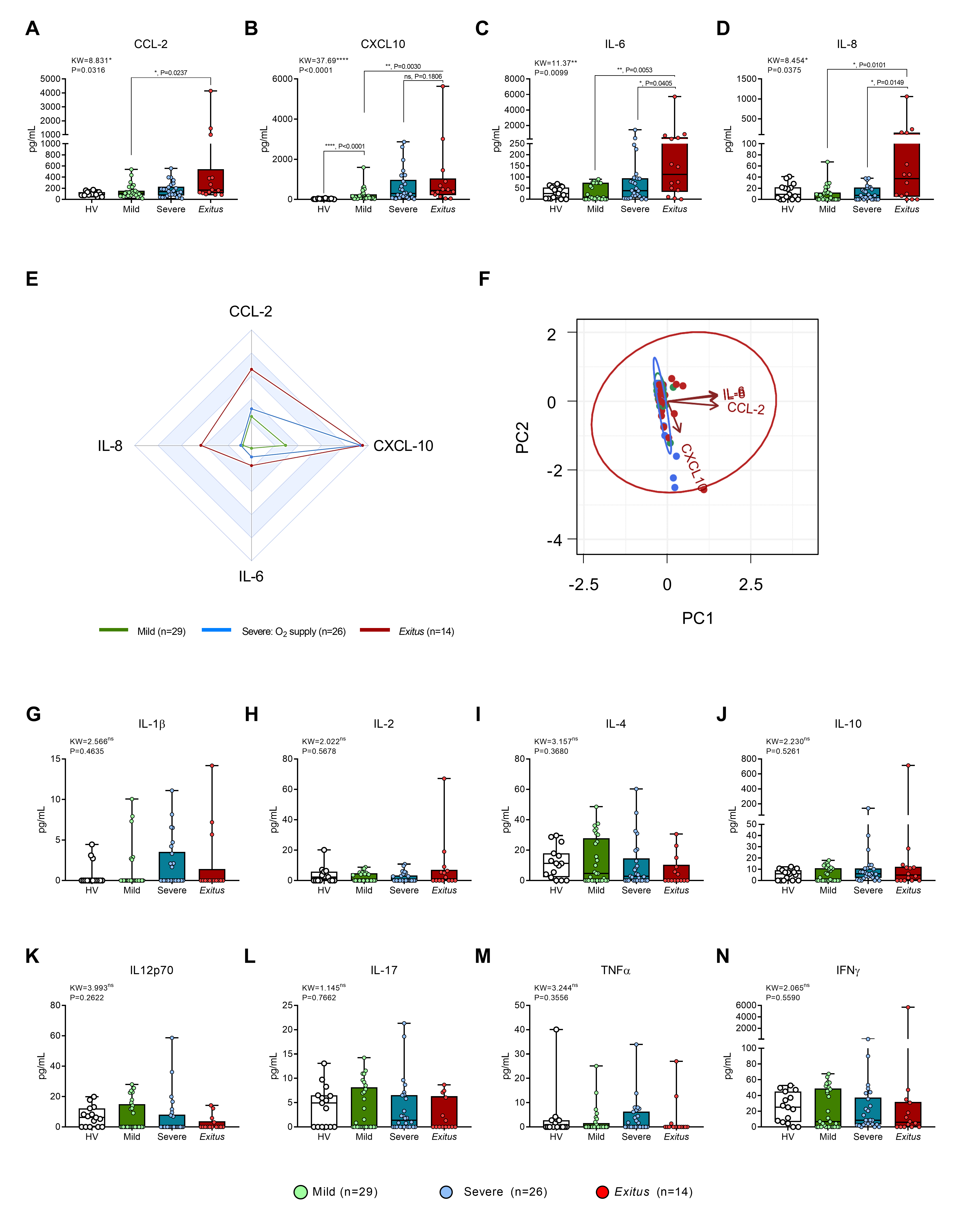
**

**Supplementary Figure 13. Plasma levels on admission of cytokines in COVID-19 patients severity groups.** Quantification of plasma CCL-2 (**A**), CXCL10 (**B**), IL-6 (**C**), and IL-8 (**D**) levels of patients with COVID-19 on admission according to evolution and their hospital needs are shown. (**E**) Radar plot of severity-associated changes in patients with COVID-19 in function of plasma CCL-2, CXCL10, IL-6, and IL-8 levels on admission is shown. (**F**) Principal component analysis (PCA) of severity-associated changes in patients with COVID-19 in function of plasma CCL-2, CXCL10, IL-6, and IL-8 levels on admission is shown. Levels of plasmatic IL-1β (**G**), IL-2 (**H**), IL-4 (**I**), IL-10 (**J**), IL-12p70 (**K**), IL-17 (**L**), TNFα (**M**) and IFNγ (**N**) in HVs and patients with COVID-19 on admission according to their severity/outcome are shown. Data are pg/mL concentrations and subgroup differences were analyzed by Kruskal-Wallis and Mann–Whitney U tests. Data represented in box-and-whisker plots (min to max). *, P < 0.05; **, P < 0.01; ****, P < 0.0001; K-W, Kruskal-Wallis-statistic.

**Supplementary Figure 14**

**
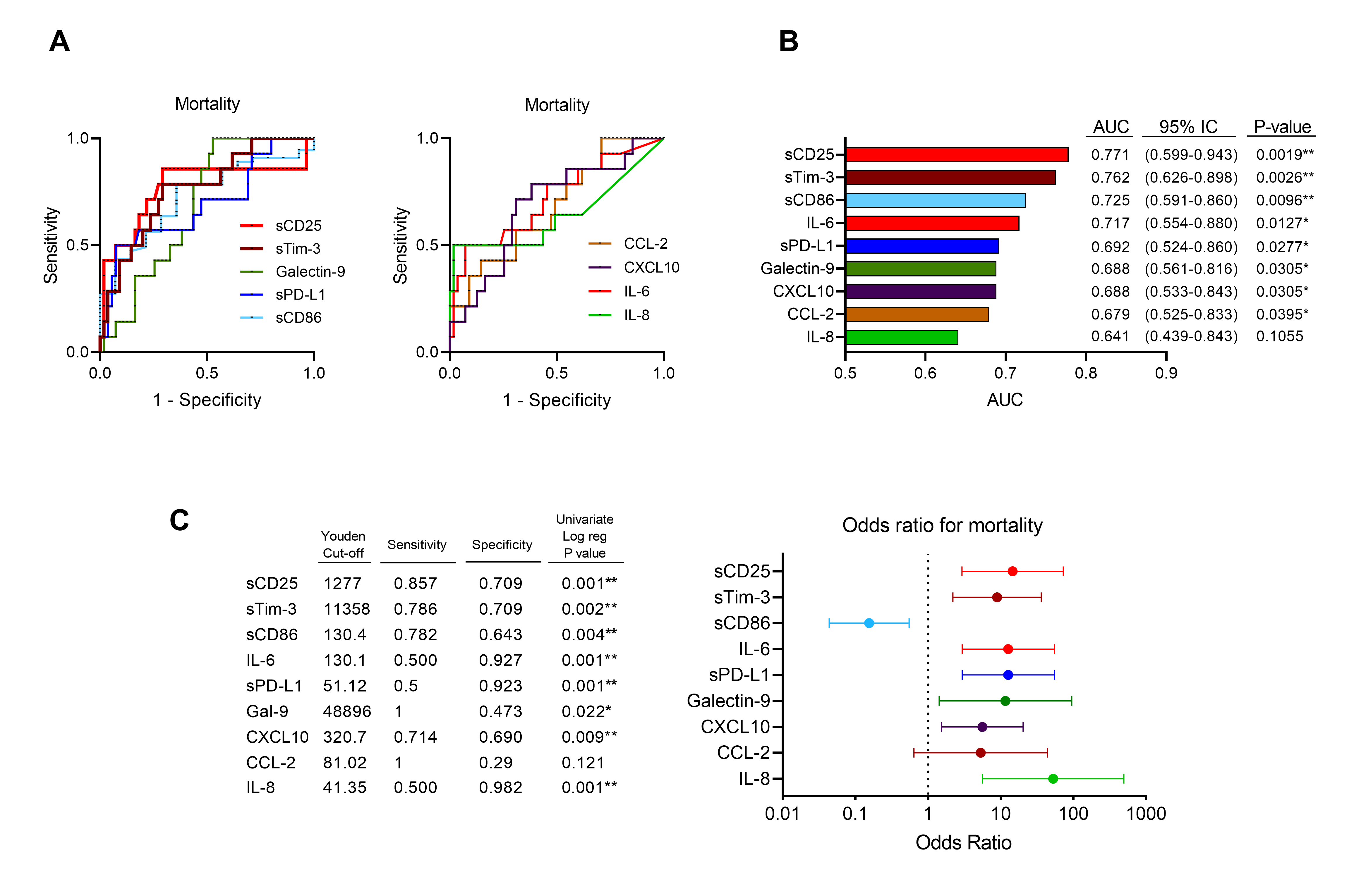
**

**Supplementary Figure 14. Plasma immune checkpoints on admission exhibit higher mortality predictive performance in COVID-19 patients than cytokines.** (**A**) ROC curves analysis of plasma immune checkpoint (left panel) and cytokine (right panel) levels of patients with COVID-19 on admission according to their outcome (survivors versus *exitus*) are shown. (**B**) Calculated AUCs of ROC curves of plasma immune checkpoint and cytokine levels of patients with COVID-19 on admission according to their outcome (survivors versus *exitus*) are shown. (**C**) Youden index-based cut-off values (left panel) and odds ratios for mortality for low *versus* high plasma immune checkpoint and cytokine levels (right panel) of COVID-19 patients on admission analyzed by univariate logistic regression. *, P < 0.05; **, P < 0.01.

**Supplementary Figure 15**

**
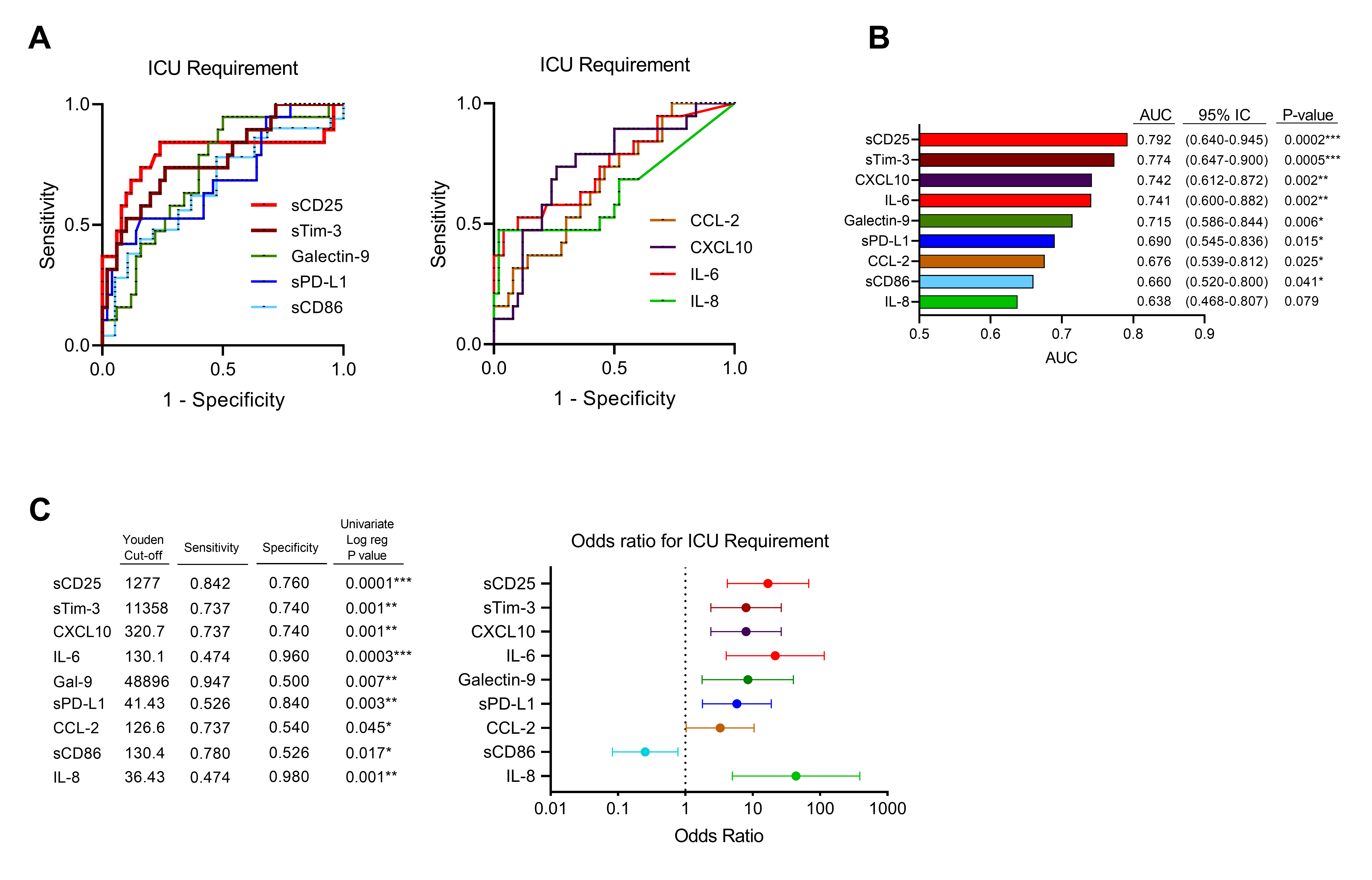
**

**Supplementary Figure 15. Plasma immune checkpoints on admission exhibit higher predictive performance of ICU requirement than cytokines in COVID-19 patients** **.** (**A**) ROC curves analysis of plasma immune checkpoint (left panel) and cytokine (right panel) levels of patients with COVID-19 on admission according to their requirement of ICU (admitted to ICU *versus* not admitted to ICU during their stay) are shown. (**B**) Calculated AUCs of ROC curves of plasma immune checkpoint and cytokine levels of patients with COVID-19 on admission according to their ICU requirement (admitted to ICU versus not admitted to ICU during their stay) are shown. (**C**) Youden index-based cut-off values for ICU requirement prediction (left panel) and odds ratios (right panel) for low *versus* high plasma immune checkpoint and cytokine levels of COVID-19 patients on admission analyzed by univariate logistic regression. *, P < 0.05; **, P < 0.01; ***, P < 0.001.
