# Supplementary material for: The immune checkpoints storm in COVID‐19: Role as severity markers at emergency department admission
Source: Clin Transl Med. 2021 Oct 18;11(10):e573. doi: 10.1002/ctm2.573 (PMC8521292; doi:10.1002/ctm2.573)
Supplement: Supplementary file 3 — Supporting Information [file CTM2-11-e573-s002.docx]

**SUPPORTING INFORMATION: SUPPLEMENTARY TABLES**

***The immune checkpoints storm in COVID-19: role as severity markers at emergency department admission***

José Avendaño-Ortiz, Roberto Lozano-Rodríguez, Alejandro Martín-Quirós, Verónica Terrón, Charbel Maroun-Eid, Karla Montalbán-Hernández, Jaime Valentín Quiroga, Miguel Ángel García-Garrido, Elena Muñoz del Val, Álvaro del Balzo-Castillo, María Peinado, Laura Gómez, Carmen Herrero-Benito, Carolina Rubio, José Carlos Casalvilla-Dueñas, Paloma Gómez-Campelo, Alejandro Pascual-Iglesias, Carlos del Fresno, Luis A. Aguirre and Eduardo López-Collazo

**SUPPLEMENTARY TABLES**

**Supplementary Table 1. Demographic and baseline characteristics of COVID-19 patients according to their hospital needs and disease severity**

| Characteristic | All patients  (n=69) | 1  (Mild, n=29) | 2  (Severe, n=26) | 3  (*Exitus,* n=14) | P-value |
| --- | --- | --- | --- | --- | --- |
| Demographic |  |  |  |  |  |
| Age – years | 62.78±18.08 | 55.45±16.87 | 62.77±15.99 | 78.00±15.32 | 0.0008 *** |
| Sex, male – n (%) | 27 (39.13) | 7 (24.14) | 12 (46.15) | 8 (57.14) |  |
| Current smoker | 3 (4.35) | 1 (4.76) | 0 (0.00) | 2 (14.29) |  |
| Coexisting disorder – n, (%) |  |  |  |  |  |
| Hypertension | 27 (39.13) | 9 (31.03) | 8 (30.77) | 10 (71.43) | 0.0214 * |
| Diabetes | 16 (23.19) | 5 (17.24) | 6 (23.08) | 5 (35.71) |  |
| Cardiovascular disease | 17 (24.64) | 7 (24.14) | 5 (19.23) | 5 (35.71) |  |
| Chronic renal disease | 9 (13.04) | 2 (6.90) | 3 (11.54) | 4 (28.57) |  |
| Obesity | 11 (15.94) | 2 (6.90) | 8 (30.77) | 1 (7.14) | 0.0326 * |
| Asthma | 3 (4.35) | 2 (6.90) | 1 (3.85) | 0 (0.00) |  |
| COPD | 10 (14.49) | 3 (10.34) | 3 (11.54) | 4 (28.57) |  |
| Oncologic disease | 5 (7.25) | 3 (10.34) | 1 (3.85) | 1 (7.14) |  |
| Immunodeficiency | 10 (14.59) | 5 (17.24) | 3 (11.54) | 2 (14.29) |  |
| Vital signs at inclusion |  |  |  |  |  |
| Glasgow Coma Scale score – n (%) | 14.59±1.30 | 14.93±0.37 | 15.00±0.00 | 13.14±2.38 | <0.0001 **** |
| Temperature – °C | 36.92±0.93 | 36.65±0.74 | 37.16±0.99 | 37.03±1.08 |  |
| Systolic arterial pressure – mm Hg | 125.26±20.44 | 127.03±20.51 | 123.35±19.85 | 125.1±22.5 |  |
| Mean arterial pressure – mm Hg | 77.13±13.41 | 77.10±14.62 | 78.77±13.57 | 74.14±10.51 |  |
| Saturation of O_2_ (SpO_2_) – (%) | 93.29±6.31 | 95.62±3.20 | 93.04±5.70 | 88.93±9.52 | 0.0033 ** |
| Fraction inspired of O_2_ (FiO_2_) – (%) | 23.23±10.03 | 21.14±0.74 | 21.81±2.48 | 30.21±21.15 | 0.0088 ** |
| SpO_2_/FiO_2_ ratio | 424.5±67.2 | 452.8±20.2 | 430.4±44.1 | 354.9±109.2 | 0.0001 *** |
| Days after onset of symptoms – days | 10.51±12.01 | 16.43±16.36 | 6.62±4.57 | 5.93±4.34 | 0.0467 * |
| Haemoglobin – g/dL | 13.77±2.05 | 13.84±1.31 | 14.15±2.59 | 12.89±2.05 |  |
| Haematocrit – (%) | 41.84±4.90 | 42.52±3.59 | 41.98±5.25 | 40.15±6.39 |  |
| White-cell counts |  |  |  |  |  |
| Counts/mm^3^ | 6867±6499 | 6443±3937 | 7232±9311 | 7069±4487 |  |
| Distribution – n, (%) |  |  |  |  |  |
| >10000 per mm^3^ | 7 (10.14) | 4 (13.79) | 1 (3.85) | 2 (14.29) |  |
| <4000 per mm^3^ | 14 (20.29) | 6 (20.69) | 4 (15.38) | 4 (28.57) |  |
| ALC |  |  |  |  |  |
| Counts/mm^3^ | 918±547 | 1236±578 | 800±386 | 479±315 | <0.0001 **** |
| Distribution – n, (%) |  |  |  |  |  |
| <1000 per mm^3^ | 41 (59.42) | 11 (37.93) | 17 (65.38) | 13 (92.86) | 0.0020 ** |
| ANC |  |  |  |  |  |
| Counts/mm^3^ | 4871±2840 | 4560±2535 | 4531±1956 | 6149±4335 |  |
| Distribution – n, (%) |  |  |  |  |  |
| >6000 per mm^3^ | 21 (30.43) | 8 (27.59) | 7 (26.92) | 6 (42.86) |  |
| AMC |  |  |  |  |  |
| Counts/mm^3^ | 320±179 | 351±151 | 308±181 | 281±225 |  |
| Distribution – n, (%) |  |  |  |  |  |
| >600 per mm^3^ | 5 (7.25) | 2 (6.90) | 2 (9.52) | 1 (7.14) |  |
| <300 per mm^3^ | 32 (48.48) | 10 (34.48) | 13 (50.00) | 9 (64.29) |  |
| Ratio N/L | 9.70±13.97 | 5.06±5.60 | 9.60±11.66 | 19.52±23.25 | 0.0005 *** |
| Platelet counts |  |  |  |  |  |
| Counts/mm^3^ | 232912±113537 | 280883±109094 | 218665±89193 | 160000±123413 | 0.0030 ** |
| Distribution – n, (%) |  |  |  |  |  |
| <150000 per mm^3^ | 14 (20.29) | 1 (3.45) | 6 (23.07) | 7 (50.00) | 0.0016 ** |
| D-dimer – ng/mL | 1828±4300 | 603±549 | 1253±1411 | 5431±8618 | 0.0002 *** |
| Creatinine – mg/dl | 1.12±1.29 | 0.86±0.91 | 1.28±1.73 | 1.38±0.92 | 0.0074 ** |
| AST – U/L | 53.55±85.09 | 30.59±21.50 | 64.38±58.13 | 81±167.80 | 0.0026 ** |
| ALT – U/L | 43.14±45.91 | 31.21±23.52 | 61.27±65.02 | 34.21±25.39 |  |
| Total bili rubin – mg/dl | 0.69±0.83 | 0.60±0.28 | 0.53±0.30 | 1.17±1.71 |  |
| LDH – U/L | 328.8±149.4 | 258.8±80.5 | 366.8±153.2 | 403.3±194.5 | 0.0012 ** |
| CRP – mg/L | 70.06±88.19 | 44.06±76.49 | 82.27±87.34 | 101.27±102.75 | 0.0042 ** |
| PCT – ng/mL | 4.70±26.78 | 0.14±0.20 | 3.23±10.04 | 16.86±57.85 | 0.0006 *** |
| Lactate – nmol/L | 1.26±0.62 | 0.97±0.22 | 1.22±0.48 | 1.94±0.87 | 0.0002 *** |
| Ferritin – ng/mL | 538.0±748.4 | 236.6±225.9 | 730.8±1014.0 | 804.0±699.4 | 0.0016 ** |
| IL-6 – pg/mL | 54.01±149.43 | 11.67±17.34 | 24.48±30.57 | 196.56±294.49 | <0.0001 **** |
| q-SOFA – n (%)^$^ | 0.81±0.73 | 0.48±0.57 | 0.77±065 | 1.57±0.65 | <0.0001 **** |
| SOFA score – n (%) | 1.45±2.33 | 0.34±1.08 | 1.00±1.39 | 4.57±2.93 | <0.0001 **** |
| Treatment during hospitalization |  |  |  |  |  |
| Corticosteroids – n (%) | 29 (42.03) | 2 (6.90) | 17 (65.38) | 10 (71.43) | <0.0001 **** |
| Tocilizumab – n (%) | 4 (5.80) | 0 (0.00) | 2 (7.69) | 2 (14.29) |  |
| Remdesivir – n (%) | 15 (21.74 | 3 (10.34) | 10 (38.46) | 2 (14.29) | 0.0311 * |
| Colchicine – n (%) | 3 (4.35) | 3 (10.34) | 0 (0.00) | 0 (0.00) |  |
| Hydroxychloroquine – n (%) | 2 (2.90) | 0 (0.00) | 1 (3.85) | 1 (7.14) |  |
| Respiratory support during hospitalization – n, (%) |  |  |  |  |  |
| Mask or nasal prongs | 26 (37.68) | 0 (0.0) | 21 (80.76) | 7 (50.00) | <0.0001 **** |
| NIV or high-flow oxygen | 6 (8.69) | 0 (0.0) | 1 (3.85) | 3 (21.43) | <0.0001 **** |
| ECMO | 2 (2.90) | 0 (0.0) | 1 (3.85) | 1 (7.14) |  |
| OTI | 6 (8.69) | 0 (0.0) | 3 (11.54) | 3 (21.43) |  |
| Sepsis during hospitalization | 8 (11.59) | 0 (0.0) | 4 (15.38) | 4 (28.57) | 0.0174 * |
| ICU admission during hospitalization | 7 (10.14) | 0 (0.0) | 3 (11.54) | 4 (28.57) | 0.0140 * |

Data are expressed as mean±SD or number (percentage).

ALC: absolute lymphocyte count, ALT: alanine aminotransferase, AMC: absolute monocyte count, ANC: absolute neutrophil count, AST: aspartate aminotransferase, CRP: C-reactive protein, COPD: chronic obstructive pulmonary disease; ECMO: Extracorporeal membrane oxygenation, ICU: intensive care unit, IL-6: interleukin 6, NIV: non-invasive ventilation, LDH: lactate dehydrogenase, OTI: Orotracheal intubation, PCT: procalcitonin, ^$^Quick Sequential Organ Failure Assessment score.

*, P< 0.05; ** P< 0.01; *** P< 0.001; **** P< 0.0001 in Chi-square or Kruskal-Wallis tests.

**Supplementary Table 2. Steps in the Wald backward stepwise regression model.**

| Step | Variables | B | SD | Wald | P-Value |
| --- | --- | --- | --- | --- | --- |
| 1 | Age  Resp Rate  SpO_2_  SpO_2_/FiO_2_  Lactate  ALC  N/L Ratio  Platelets  D-Dimer  qSOFA  sCD25  sCD86  sTim3  AHT | 1.736  6.247  -.682  -.596  34.023  .023  -1.630  .000  .007  -42.214  .023  -.155  -.002  47.697 | 525.31  786.20  525.31  86.98  11612.46  6.21  614.03  .03  2.37  5683.60  2.48  53.07  .41  13230.06 | .000011  .000063  .000002  .000047  .000009  .000014  .000007  .000085  .000009  .000055  .000088  .000009  .000025  .000013 | .997  .994  .999  .995  .998  .997  .998  .993  .998  .994  .993  .998  .996  .997 |
| 2 | Age  Resp Rate  SpO_2_/FiO_2_  Lactate  ALC  N/L Ratio  Platelets  D-Dimer  qSOFA  sCD25  sCD86  sTim3  AHT | 2.353  7.379  -.618  49.393  .021  -2.228  .000  .009  -49.837  .026  -.180  -.002  46.264 | 415.33  743.35  103.65  11985.01  8.81  417.70  .03  2.36  7761.39  2.16  64.77  .51  5811.95 | .00003  .00010  .00004  .00002  .00001  .00003  .00012  .00001  .00004  .00014  .00001  .00002  .00006 | .995  .992  .995  .997  .998  .996  .991  .997  .995  .990  .998  .997  .994 |
| 3 | Age  Resp Rate  SpO_2_/FiO_2_  Lactate  N/L Ratio  Platelets  D-Dimer  qSOFA  sCD25  sCD86  sTim3  AHT | 1.985  7.768  -.649  49.319  -3.153  .000  .012  -51.513  .026  -.172  -.003  49.220 | 535.99  695.92  98.04  10496.15  343.88  .04  2.25  12891.68  2.37  106.77  .83  6729.72 | .00001  .00012  .00004  .00002  .00008  .00009  .00003  .00002  .00012  .00000  .00001  .00005 | .997  .991  .995  .996  .993  .993  .996  .997  .991  .999  .998  .994 |
| 4 | Age  Resp Rate  SpO_2_/FiO_2_  Lactate  N/L Ratio  Platelets  D-Dimer  qSOFA  sCD25  sTim3  AHT | 2.989  11.710  -1.041  78.595  -4.264  .000  .018  -58.669  .038  -.005  56.954 | 217.80  694.83  71.69  11747.72  576.08  .03  1.48  4148.25  2.10  .30  8617.63 | .00019  .00028  .00021  .00004  .00005  .00023  .00014  .00020  .00032  .00022  .00004 | .989  .987  .988  .995  .994  .988  .991  .989  .986  .988  .995 |
| 5 | Age  Resp Rate  SpO_2_/FiO_2_  Lactate  N/L Ratio  Platelets  D-Dimer  qSOFA  sCD25  sTim3 | 57.289  250.343  -7.545  1655.310  -12.890  -.010  .326  -245.735  .785  -.110 | 209.85  921.95  27.48  6153.20  49.76  .04  1.20  893.79  2.89  .40 | .07453  .07373  .07535  .07237  .06708  .07374  .07334  .07559  .07377  .07366 | .785  .786  .784  .788  .796  .786  .787  .783  .786  .786 |
| 6 | Age  Resp Rate  SpO_2_/FiO_2_  Lactate  Platelets  D-Dimer  qSOFA  sCD25  sTim3 | 165.614  695.153  -23.278  3117.222  -.027  .726  -612.891  1.920  -.279 | 433.14  1821.97  60.62  8216.79  .07  1.90  1592.52  5.03  .73 | .14619  .14557  .14743  .14392  .14554  .14524  .14811  .14555  .14552 | .702  .703  .701  .704  .703  .703  .700  .703  .703 |
| 7 | Age  Resp Rate  SpO_2_/FiO_2_  Platelets  D-Dimer  qSOFA  sCD25  sTim3 | .864  2.434  -.186  .000  .002  -7.144  .007  -.001 | .47  1.69  .10  .0001  .0010  4.1095  .0043  .0006 | 3.312  2.062  3.028  2.370  3.114  3.022  2.655  2.233 | .069  .151  .082  .124  .078  .082  .103  .135 |
| 8 | Age  SpO_2_/FiO_2_  Platelets  D-Dimer  qSOFA  sCD25  sTim3 | .18419  -.04920  -.00001  .00035  -.05193  .00107  -.00004 | .0818  .0243  .0000  .0002  1.3192  .0007  .0001 | 5.075  4.090  1.347  4.319  .002  2.515  .295 | .024  .043  .246  .038  .969  .113  .587 |
| 9 | Age  SpO_2_/FiO_2_  Platelets  D-Dimer  sCD25  sTim3 | .18279  -.04890  -.00001  .00035  .00107  -.00004 | .0735  .0231  .0000  .0002  .0007  .0001 | 6.191  4.475  1.522  4.459  2.550  .307 | .013  .034  .217  .035  .110  .580 |
| 10 | Age  SpO_2_/FiO_2_  Platelets  D-Dimer  sCD25 | .18514  -.04493  -.00001  .00032  .00083 | .0727  .0205  .0000  .0001  .0005 | 6.478  4.824  1.393  4.963  3.247 | .011  .028  .238  .026  .072 |
| 11 | Age  SpO_2_/FiO_2_  D-Dimer  sCD25 | .18283  -.03876  .00035  .00081 | .0703  .0159  .0001  .0005 | 6.766  5.952  7.440  2.964 | .009  .015  .006  .085 |
| 12 | Age  SpO_2_/FiO_2_  D-Dimer  sCD25  sCD86 | .18381  -.03345  .00034  .00113  -.01682 | .0749  .0158  .0002  .0006  .0096 | 6.025  4.500  4.337  3.080  3.068 | .014  .034  .037  .079  .080 |

AHT, arterial hypertension; Resp rate, respiratory rate; SpO_2_, oxygen saturation; SpO_2_/FiO_2_, peripheral blood oxygen saturation to fraction of inspired oxygen ratio; ALC, absolute lymphocyte counts; N/L Ratio, neutrophil to lymphocyte ratio; AST, aspartate transaminase; qSOFA, quick sequential organ failure assessment score.

B weight coefficient; SD, standard deviation of B; Wald, Wald statistic

**Supplementary Table 3. Demographics and baseline characteristics of patients included in the validation cohort**

|  | All patients  (n=166) | 1  (Mild, n=66) | 2  (Severe, n=85) | 3  (*Exitus*, n=15) | P-value |
| --- | --- | --- | --- | --- | --- |
| Age – years | 58.11±15.25 | 50.47±15.48 | 61.66 ±12.32 | 71.67±12.92 | <0.0001 **** |
| Sex, male – n (%) | 98 (59.04) | 28 (42.42) | 63 (74.12) | 7 (46.67) | 0.0003 *** |
| Smoking history – n, (%) |  |  |  |  |  |
| Never smoked | 139 (83.73) | 63 (95.46) | 63 (74.12) | 13 (86.67) | 0.0019 ** |
| Former smoker | 22 (13.25) | 1 (1.52) | 20 (23.53) | 1 (6.67) | 0.0003 *** |
| Current smoker | 5 (3.01) | 2 (3.03) | 2 (2.35) | 1 (6.67) |  |
| Addicted to alcohol – n, (%) | 7 (4.22) | 0 (0.00) | 7 (8.24) | 0 (0.00) | 0.0307 * |
| Saturation of O_2_ (SpO_2_) – % | 93.25±6.59 | 95.97±4.02 | 92.47±5.49 | 85.67±12.47 | <0.0001 **** |
| Fraction inspired of Oxygen (FiO_2_) – (%) | 23.23±10.03 | 21.14±0.74 | 21.81±2.48 | 30.21±21.15 | 0.0088 ** |
| 21 | 143 (86.14) | 66 (100.00) | 71 (83.53) | 6 (40.00) | <0.0001 **** |
| 22 to 25 | 5 (3.01) | 0 0.00) | 4 (4.71) | 1 (6.67) |  |
| 26 to 40 | 11 (6.63) | 0 (0.00) | 9 (10.59) | 2 (13.33) | 0.0190 * |
| 60 to 100 | 7 (4.22) | 0 (0.00) | 1 (1.18) | 6 (40.00) | <0.0001 **** |
| SpO_2_/FiO_2_ | 418.3±83.8 | 457.0±19.2 | 419.1±59.2 | 243.7±142.5 | <0.0001 **** |
| D-dimer – ng/mL | 1019±1444 | 615±572 | 1138±1589 | 2121±2398 | <0.0001 **** |
| Coexisting disorder – n, (%) |  |  |  |  |  |
| Hypertension | 57 (34.34) | 14 (21.21) | 34 (40.00) | 9 (60.00) | 0.0271 * |
| Diabetes | 27 (16.27) | 5 (7.58) | 18 (21.18) | 4 (26.67) | 0.0417 * |
| Cardiovascular disease | 15 (9.04) | 3 (4.45) | 11 (12.94) | 1 (6.67) |  |
| Chronic renal disease | 4 (2.41) | 0 (0.00) | 2 (2.35) | 2 (13.33) | 0.0098 ** |
| Obesity | 15 (9.04) | 3 (4.45) | 12 (14.12) | 0 (0.00) |  |
| Asthma | 13 (7.83) | 4 (6.06) | 9 (10.59) | 0 (0.00) |  |
| COPD^$^ | 14 (8.43) | 4 (6.06) | 8 (9.41) | 2 (13.33) |  |
| Oncologic disease | 11 (6.63) | 3 (4.45) | 11 (12.94) | 0 (0.00) |  |
| Immunodeficiency | 14 (7.19) | 5 (7.58) | 5 (5.88) | 4 (26.67) | 0.0268 * |
| Respiratory support during hospitalization – n, (%) |  |  |  |  |  |
| Mask or nasal prongs | 72 (43.37) | 0 (0.0) | 66 (77.64) | 6 (40.00) | <0.0001 **** |
| NIV or high-flow oxygen | 5 (3.01) | 0 (0.0) | 3 (3.53) | 2 (13.33) | 0.0224* |
| ECMO | 7 (4.22) | 0 (0.0) | 5 (5.88) | 2 (13.33) | 0.0373* |
| OTI | 16 (9.63) | 0 (0.0) | 11 (12.94) | 5 (33.33) | 0.001*** |
| Sepsis during hospitalization | 19 (11.44) | 0 (0.0) | 14 (16.47) | 5 (33.33) | 0.001*** |
| ICU admission during hospitalization | 22 (13.25) | 0 (0.0) | 16 (18.82) | 6 (40.00) | <0.0001 **** |

^$^ Chronic obstructive pulmonary disease, ECMO: Extracorporeal membrane oxygenation, ICU: intensive care unit, 6, NIV: non-invasive ventilation, OTI: Orotracheal intubation,

*, P< 0.05; ** P< 0.01; *** P< 0.001; **** P< 0.0001 in Chi-square or Kruskal-Wallis tests.

**Supplementary Table 4.** **List of all fluorochrome-conjugated monoclonal antibodies for flow cytometry analysis and monoclonal antibodies**

|  | Fluorochrome | Source | Clone | Reference |
| --- | --- | --- | --- | --- |
| CD3 | Spark Blue 550 | Biolegend | SK7 | Cat# 344852 |
| CD4 | BV570 | Biolegend | RPA-T4 | Cat# 300534 |
| CD8 | Pacific Orange | ThermoFisher Scientific | 3B5 | Cat# MHCD0830 |
| CD137 (4-1BB) | BV786 | BD Bioscience | 4B4-1 | Cat# 741000 |
| CD152 (CTLA-4) | Alexa Fluor 647 | Biolegend | BNI3 | Cat# 369626 |
| CD223 (LAG-3) | APC-R700 | BD Bioscience | T47-530 | Cat# 565774 |
| CD276 (PD-1) | BUV563 | BD Bioscience | 7-517 | Cat# 748380 |
| CD366 (Tim-3) | APC eFluor 780 | ThermoFisher Scientific |  | Cat# 47-3109-42 |

|  | Isotype | Clone | Source/Reference |
| --- | --- | --- | --- |
| Basiliximab (anti-CD25) | Chimeric (Human/Murine) IgG1_κ_ | - | Novartis |
| Pembrolizumab (anti-PD-1) | Chimeric (Human/Murine) IgG4_κ_ | - | Merck |
| CD336 (Tim-3) | Rat IgG2a _κ_ | RMT3-23 | Biolegend (Cat# 119732) |
| Galectin-9 | Mouse IgG1_κ_ | 9M1-3 | Biolegend (Cat# 348926) |
