# Supplementary material for: The immune checkpoints storm in COVID‐19: Role as severity markers at emergency department admission
Source: Clin Transl Med. 2021 Oct 18;11(10):e573. doi: 10.1002/ctm2.573 (PMC8521292; doi:10.1002/ctm2.573)
Supplement: Supplementary file 4 — Supporting Information [file CTM2-11-e573-s001.docx]

**SUPPORTING INFORMATION: STROBE STATEMENT**

***The immune checkpoints storm in COVID-19: role as severity markers***  ***at emergency department admission***

José Avendaño-Ortiz, Roberto Lozano-Rodríguez, Alejandro Martín-Quirós, Verónica Terrón, Charbel Maroun-Eid, Karla Montalbán-Hernández, Jaime Valentín Quiroga, Miguel Ángel García-Garrido, Elena Muñoz del Val, Álvaro del Balzo-Castillo, María Peinado, Laura Gómez, Carmen Herrero-Benito, Carolina Rubio, José Carlos Casalvilla-Dueñas, Paloma Gómez-Campelo, Alejandro Pascual-Iglesias, Carlos del Fresno, Luis A. Aguirre and Eduardo López-Collazo

**STROBE Statement**

Checklist of items that should be included in reports of observational studies

| **Section/Topic** | **Item No** | **Recommendation** | **Reported on** |
| --- | --- | --- | --- |
| **Title and summary** | 1 | The immune checkpoints storm in COVID-19: role as severity markers at emergency department admission. | Page 1 |
|  |  | Immune checkpoints (ICs) and their ligands as severity biomarkers and hospital requirements in COVID-19. | 9^th^ paragraph |
| **Introduction** | | | |
| Background/rationale | 2 | Evidence has suggested that SARS-CoV-2 infection impacts the immune system and takes advantage of several mechanisms and pathways, including cytokine storm, blood leukocyte apoptosis, and T cell exhaustion. Although there is evidence of T cell activation in these patients, numerous studies have found how polyfunctionality or cytotoxicity decreases in COVID-19 patients. | 1^st^ paragraph |
| Objectives | 3 | The main objective is to evaluate the potential role of ICs as early biomarkers of severity and outcome in patients with COVID-19. In addition, we studied the possible link between COVID-19 associated T cell exhaustion and the levels of inhibitory immune checkpoint ligands. | 1^st^ to 5^th^ paragraphs |
| **Methods** | | | |
| Study design | 4 | As a discovery cohort, blood samples from 69 COVID-19 patients were prospectively collected on admission and until discharge or *exitus*. Patients were classified according to the oxygen requirement during their stay. Prospectively-collected plasma samples from COVID-19 patients (n=166) recruited from January 11 to March 26, 2021 in La Paz University Hospital were used as a validation cohort. | 2nd paragraph and Supporting information |
| Setting | 5 | Blood samples from 69 COVID-19 patients were collected from April 28 to September 21, 2020 at the emergency department at La Paz University Hospital. | 2nd paragraph and Supporting information |
| Participants | 6 | A total of 235 individuals with COVID-19 (69 for prospective discovery cohort, 166 for retrospective validation cohort). Fifteen healthy volunteers were recruited as controls. | 2nd paragraph and Supporting information |
| Variables | 7 | - Clinical: Maximum health care requirement during their stay, Outcome, Age, Sex, Comorbidities, Temperature, Heart Rate, Respiratory rate, Oxygen saturation (SpO_2_), fraction of inspired oxygen (FiO_2_), peripheral blood oxygen saturation to fraction of inspired oxygen ratio (SpO_2_/FiO_2_), lactate, absolute leukocyte counts, absolute lymphocyte counts, absolute neutrophil counts, absolute monocyte counts, neutrophil to lymphocyte ratio, platelets, ferritin, D-Dimer, creatinine, aspartate transaminase, alanine transaminase, lactate dehydrogenase, c-reactive protein, procalcitonin, quick sequential organ failure assessment score (qSOFA).  - Laboratory: soluble ICs (sCD25, sCD137, sCD86, sCTLA-4, sPD-L1, sPD-1,sTim-3, sLAG-3 and Galectin-9), cytokines and chemokines (IL-1β, IL-2, IL-4, IFN-γ, TNF-α, CCL-2, CXCL10, IL-6, IL-8, IL-10, IL-12p70 and IL-17A. | Table 1, Supporting information |
| Data sources/measurement | 8* | Data from our laboratory and clinical follow up. | Supporting information |
| Bias | 9 | N/A |  |
| Study size | 10 | Not predefined. Following previous similar studies. |  |
| Quantitative variables | 11 | For quantitative variables data are presented as means and standard deviations. Differences between groups were evaluated using the Mann Whitney U-test for two groups comparison and Kruskal-Wallis for multiple groups comparisons. Associations among soluble cytokines and immune checkpoints with clinical parameters or proliferation levels of CD4^+^ and CD8^+^ cells were evaluated by Spearman’s correlation analysis. Wald backward stepwise regression was used for Score estimation. | Supporting information |
| Statistical methods | 12 | Differences between groups were evaluated with the use of a chi-squared test for categorical variables, Student’s t-test for comparison of quantitative variables between two groups. Logistic regression model for mortality prediction was performed by Wald backward stepwise regression, including age, previous diagnosis of hypertension, respiratory rate, SpO_2_, SpO_2_/FiO_2_ ratio, lactate, ALC, neutrophils/lymphocytes ratio, platelets, D-Dimer, q-SOFA, sCD25, sTim-3 and sCD86. The final model after the twelve steps score can be calculated with the following formula: Score = 0.18381 x Age - 0.3345 x SpO2/FiO2 + 0.00034 x D-Dimer + 0.00113 x sCD25 - 0.01682 x sCD86. Coefficients and Odds-ratio (OR) and P-values of the final model are shown in Table 4. All optimal cut-off values were estimated by the Youden index. P-values of less than 0.05 were considered to indicate statistical significance. All p-values are 2-sided, and 95% confidence intervals (95% CI) are also presented. Statistical analyses were conducted using Prism 8.0 (GraphPad) and SPSS version 23 (IBM) software. Specifically statistical information is included in figure legends | Supporting information |

| **Section/Topic** | **Item No** | **Recommendation** | **Reported on** |
| --- | --- | --- | --- |
| **Results** | | | |
| Participants | 13* | A total of 235 individuals with COVID-19 (69 for prospective discovery cohort, 166 for retrospective validation cohort). Fifteen healthy volunteers were recruited as controls. | 2^nd^ and 8^th^ paragraphs |
| Descriptive data | 14* | Descriptive data from the groups of individuals are summarised in Supplementary tables 1 and 3 | Supplementary tables 1 and 3 |
| Outcome data | 15* | Oxygen requirement during their stay (independently if it was mask/nasal prongs, non-invasive ventilation or hifh-flow oxygen or OT intubation or ECMO), and death (28-day mortality according WHO U07.1 code) | 2^nd^ paragraph and Supporting information. |
| Main results | 16 | Five ICs showed differences between patients who recovered and patients who subsequently died: sCD25, sTim-3, Galectin-9, sPD-L1, and sCD86. ROC analyses indicated that ICs are better predictors of patient evolution than levels of cytokines and other biomarkers. A binary logistic regression analysis identified sCD86, sPD-L1, sTim-3, and age as independent predictors of in-hospital mortality from COVID-19. The relevance of the calculated score was confirmed in a retrospectively obtained validation cohort (sensitivity 96%; specificity 93%; odds ratio [OR] 338.3 [95% CI 41.88-3573]; p<0.0001). | 2^nd^ to 8^th^ paragraphs |
| Other analyses | 17 | Other analyses and results are explained in the Supporting information | Supporting information |
| **Discussion** | | | |
| Key results | 18 | Patients who subsequently died showed higher sCD25, sTim-3, Galectin-9, and sPD-L1, and lower sCD86 levels than patients who recovered from the disease. Furthermore, sCD25, sTim-3, Galectin-9, and sPD-L1 also showed a positive correlation between their plasma concentrations and disease severity.  Furthermore, the score obtained from our Wald backward stepwise model, including sCD86, sCD25, age, D-Dimer, and SpO_2_/FiO_2_ showed a strong performance, predicting mortality not only in the discovery cohort but also in the validation cohort. | 2^nd^ to 9^th^ paragraphs |
| Limitations | 19 | Our data appear to indicate that ICs are robust biomarkers for COVID-19 severity. Nevertheless, a deeper and larger longitudinal study would be necessary to be able to conclude this finding definitively. Additionally, the development of a quicker and easier kit for IC quantification would be necessary in order to include this technique in the hospital routine. | Study limitations section in Supporting information |
| Interpretation | 20 | Higher levels of sCD25, sTim-3, Galectin-9, sPD-L1, and lower levels of sCD86 are associated with critical COVID-19 outcome. In the validation cohort our scores, estimated from logistic regression from clinical routine data and IC levels, performed well at identifying on admission those patients who subsequently died of required endotracheal intubation. These data suggest that successful ICs could have a role as biomarkers in COVID-19 and be a good candidate for SARS-CoV-2 infection monitoring. | 2^nd^ to 9^th^ paragraphs |
| Generalizability | 21 | A larger multi-center longitudinal study would be necessary. | Study limitations section in Supporting information |
| **Other Information** | | | |
| Funding | 22 | This study was supported by grants from Banco Santander, Reale Seguros, Fundación Mutua Madrileña, Fundación Uria, Fundación Caixa and Ayuntamiento de Madrid. Partially supported by fondos de Investigación Sanitarias and Fondos FEDER (PI18/00148 and PIE15/00065) to ELC. | Page 7 |

**Give information separately for cases and controls in case-control studies and, if applicable, for exposed and unexposed groups in cohort and cross-sectional studies.*

**Note:** An Explanation and Elaboration article discusses each checklist item and gives methodological background and published examples of transparent reporting. Information on the STROBE Initiative is available at www.strobe-statement.org.
